# Associations between reported atrial fibrillation burden and cardiac substructure dosimetric variables in patients receiving thoracic radiotherapy for malignancies involving the thorax: a systematic review and meta-analysis

**DOI:** 10.3389/fonc.2026.1910673

**Published:** 2026-09-10

**Authors:** Rui Li, Yingkai Chen, Qiao Li, Nengping Zhu, Yong Chen, Kai Jin, Jie Chen, Sheng Guo, Xuefen Liu, Yue Li

**Affiliations:** 1 Department of Oncology, The People’s Hospital of Rongchang District, Chongqing, China; 2 Department of Thoracic Surgery, The People’s Hospital of Rongchang District, Chongqing, China; 3 Department of Cardiology, The People’s Hospital of Rongchang District, Chongqing, China; 4 Department of Neurosurgery, The People’s Hospital of Rongchang District, Chongqing, China

**Keywords:** atrial fibrillation, cardiac substructure, meta-analysis, pulmonary vein, radiation-associated cardiac injury, sinoatrial node, systematic review, thoracic radiotherapy

## Abstract

**Introduction:**

Thoracic radiotherapy is integral to multimodal treatment for malignancies involving the thorax, but the literature on the burden of reported atrial fibrillation (AF) after thoracic radiotherapy and its relationship with cardiac substructure dose exposure has not been systematically synthesized.

**Methods:**

We searched PubMed, Embase, the Cochrane Library, and Web of Science through May 17, 2026, for studies that reported AF incidence or comparative/dosimetric associations among adults receiving thoracic radiotherapy for malignancies. Pooled incidence was estimated using logit-transformed random-effects proportion meta-analysis. Because cardiac structures, dose metrics, cutoffs, statistical models, and AF definitions differed across studies, dosimetric evidence was summarized using structured tables and evidence plots.

**Results:**

Ten studies were included. Eight studies contributed nine cohort-level estimates to the incidence synthesis, including 3,192 patients, of whom 293 developed study-defined reported AF. The pooled incidence across all eligible cohorts was 9.1% (95% confidence interval (CI), 6.3%–12.9%; I²=90.0%; prediction interval, 2.5%–28.5%). After the exclusion of two esophageal cancer cohorts with substantial perioperative confounding, the main analysis included seven cohorts with a total of 2,766 patients, among whom 217 developed reported AF; the pooled incidence was 7.6% (95% CI, 5.6%–10.3%; I²=80.3%; prediction interval, 2.9%–18.8%). Nine reports described associations between cardiac or cardiac substructure radiation doses and AF risk. Adjusted findings suggested candidate signals involving the pulmonary veins, atria, sinoatrial node, and selected whole-heart dose metrics, although the findings were heterogeneous and unsuitable for quantitative pooling.

**Conclusion:**

Study-defined reported AF after thoracic radiotherapy represents a clinically relevant burden. Current evidence suggests candidate dosimetric regions involving the pulmonary veins, atria, and sinoatrial node, with selected whole-heart metrics showing signals. However, the evidence remains insufficient to support causal inference or clinical dose constraints. Prospective multicenter studies using standardized AF definitions, cardiac substructure delineation, and individual-patient data are needed to validate these signals.

## 1 Introduction

According to GLOBOCAN 2022 data, lung cancer remains the leading cause of cancer-related death worldwide, and esophageal cancer is a major contributor to global cancer mortality. Because lung cancer and esophageal cancer are representative malignancies among patients receiving thoracic radiotherapy and because the treated population is substantial, treatment-related late toxicities have considerable clinical relevance (1).

Radiotherapy is a key component of curative-intent or multimodal treatment for a range of thoracic malignancies, particularly unresectable locally advanced non-small cell lung cancer and locally advanced esophageal cancer. For unresectable stage III non-small cell lung cancer, platinum-based concurrent chemoradiotherapy forms the backbone of standard treatment, and consolidation durvalumab after chemoradiotherapy further improves long-term survival and disease control (2, 3). For selected patients with locally advanced esophageal or esophagogastric junction cancer who are candidates for surgery, long-term follow-up evidence supports the use of neoadjuvant chemoradiotherapy followed by surgery as an important multimodal treatment pathway; this benefit has been further confirmed by long-term randomized evidence in patients with locally advanced esophageal squamous cell carcinoma (4, 5).

Research on radiation-associated cardiac injury has traditionally focused on clinical outcomes such as ischemic heart disease, heart failure, pericardial disease, valvular heart disease, and composite cardiac events. Recent systematic reviews and dosimetric studies have shown that the mean heart dose and coronary artery dose received during breast cancer radiotherapy are associated with subsequent cardiovascular morbidity and ischemic heart disease mortality; in patients with non-small cell lung cancer and other malignancies receiving thoracic radiotherapy, doses to the cardiac base, the whole heart, and selected cardiac substructures have also been associated with cardiac events or survival outcomes (6–9).

Atrial fibrillation is among the most common arrhythmias encountered in clinical practice and is closely associated with stroke, heart failure, recurrent hospitalization, impaired quality of life, and increased mortality. In patients with cancer, atrial fibrillation typically arises from a multifactorial substrate that may include advanced age, conventional cardiovascular risk factors, inflammation, hypoxia, surgery, chemotherapy, immune checkpoint inhibitors, autonomic imbalance, and direct or indirect cardiac injury (10, 11). Compared with other treatment-related factors, atrial fibrillation after thoracic radiotherapy may have more distinct anatomic and dosimetric characteristics because incidental irradiation of the atrial myocardium, pulmonary vein ostia, sinoatrial node region, and adjacent conduction tissues is influenced by tumor location, target volume, and radiotherapy plan geometry.

Although evidence in this area is accumulating, direct comparison of atrial fibrillation after thoracic radiotherapy across studies remains challenging. Previous cohorts have differed substantially in terms of cancer type, treatment setting, handling of preexisting atrial fibrillation, methods of atrial fibrillation ascertainment, event severity, follow-up duration, perioperative confounding, and handling of competing risks; moreover, dosimetric reports have used heterogeneous cardiac structures and dose–volume metrics, limiting quantitative pooling of dose–response effects. Accordingly, this systematic review and meta-analysis aimed to quantify the study-defined reported incidence of atrial fibrillation after thoracic radiotherapy and to systematically synthesize evidence linking cardiac and cardiac substructure doses to the risk of atrial fibrillation.

## 2 Methods

### 2.1 Study design and guidelines

This systematic review and meta-analysis was conducted and reported in accordance with the PRISMA 2020 statement and the MOOSE recommendations for meta-analyses of observational studies (12, 13). Before full-text screening, data extraction, and statistical analysis, the research team developed an internal study protocol that specified the research questions, eligibility criteria, outcomes, data extraction items, and analytic strategies; however, this protocol was not prospectively registered or publicly available. Because this study used aggregated data from published studies and did not involve individual patient-level information, ethics approval and informed consent were not required.

### 2.2 Literature search strategy

Two reviewers systematically searched PubMed, Embase, the Cochrane Library, and Web of Science from inception to May 17, 2026. The search strategy was structured around three core concepts: thoracic malignancies, thoracic radiotherapy exposure, and atrial fibrillation or arrhythmia outcomes. The main search terms included “atrial fibrillation,” “new-onset atrial fibrillation,” and “atrial arrhythmia,” combined with radiotherapy-related terms such as “radiotherapy,” “radiation therapy,” and “thoracic radiotherapy,” as well as tumor-related terms such as “lung cancer,” “non-small cell lung cancer,” “small cell lung cancer,” “esophageal cancer,” “mediastinal,” and “lymphoma.” To improve retrieval of cardiac dosimetry studies, supplementary searches were performed using terms related to cardiac dose assessment, including “cardiac substructure,” “heart dose,” “mean heart dose,” “cardiac dose,” “sinoatrial node,” “pulmonary vein,” “dosimetry,” and related dose–volume metrics. The search strategies were adapted for each database according to its syntax; the representative PubMed search strategy is provided in **Supplementary Table 1**. The completed PRISMA 2020 checklist is provided in **Supplementary Table 2**. The reference lists of the included studies and relevant reviews were also manually screened to identify additional eligible studies.

### 2.3 Eligibility criteria

This review included studies of adult patients with malignancies who received thoracic radiotherapy. Eligible treatment settings included definitive radiotherapy, concurrent chemoradiotherapy, neoadjuvant chemoradiotherapy, postoperative radiotherapy, or mixed thoracic radiotherapy cohorts. Eligible studies were required to report at least one of the following: the number of atrial fibrillation events with an analyzable denominator; the incidence of newly diagnosed, reported, or study-defined atrial fibrillation after treatment; comparative associations between radiation exposure and atrial fibrillation risk; or associations between whole-heart or cardiac substructure dose and atrial fibrillation risk. Eligible study designs included cohort studies, population-based database studies, registry-based studies, and secondary analyses of existing clinical data.

Reviews, commentaries, case reports, animal studies, nonthoracic radiotherapy studies, reports available only as conference abstracts or study protocols without extractable full-text data, and studies from which atrial fibrillation-specific outcomes could not be extracted were excluded. Studies that reported only composite arrhythmia endpoints without separately extractable atrial fibrillation events were not included in the quantitative incidence synthesis; however, if such studies provided comparative or dosimetric information related to atrial fibrillation, they were considered for narrative or structured evidence synthesis.

### 2.4 Data extraction

Two reviewers independently extracted data using a prespecified standardized data extraction form. Discrepancies were resolved by discussion and, when necessary, adjudicated by a third reviewer. The extracted items included the first author, publication year, country or region, study design, data source, cancer type and treatment setting, original cohort size, atrial fibrillation risk-set denominator, number of atrial fibrillation events, definition and ascertainment source of atrial fibrillation, event severity, handling of preexisting atrial fibrillation, follow-up duration, AF surveillance schedule or observation window, adjudication process, and role of each study in the evidence synthesis. Overall follow-up and AF-specific surveillance were also recorded separately when distinguishable, and unreported items were recorded as such.

For dosimetric studies, the extracted variables included the whole heart or cardiac substructure evaluated, the imaging or planning dataset used, the delineation method, operator expertise and blinding, contour verification or reproducibility procedures, dose–volume metrics, the operational definition of maximum or near-maximum dose, dose units, cutoff values, effect-size measures, adjusted estimates, 95% confidence intervals, P values, and major covariates included in multivariable models. When multiple dose metrics were reported within the same study, all multivariable-adjusted results directly related to atrial fibrillation outcomes were extracted. In the structured synthesis, emphasis was placed on dosimetric signals that were atrial fibrillation specific, adjusted, and clinically interpretable. Detailed AF ascertainment and surveillance characteristics and cardiac substructure delineation and dosimetric methods are summarized in **Supplementary Tables 3** and **4**, respectively.

### 2.5 Eligibility for synthesis and data preparation

The appropriate synthesis approach for each study was determined according to the cancer type, radiotherapy setting, definition of atrial fibrillation, handling of preexisting atrial fibrillation, event severity, perioperative or surgery-related confounding, availability of extractable event counts and denominators, and reported dosimetric metrics. Quantitative incidence synthesis was restricted to studies or cohorts with separately extractable atrial fibrillation event counts and analyzable denominators. The main incidence analysis was restricted to cohorts without substantial perioperative or surgery-related confounding. Studies primarily involving esophageal cancer treated with neoadjuvant chemoradiotherapy followed by surgery or trimodal therapy, in which atrial fibrillation may be substantially influenced by perioperative factors, were included only in extended or exploratory analyses. When a study reported multiple independent cohorts or subgroups, data were extracted separately at the cohort or subgroup level. When potential population overlap was identified, double counting within the same pooled analysis was avoided.

Studies evaluating comparative associations between radiation exposure and atrial fibrillation risk were included in the narrative synthesis. Studies reporting associations between cardiac or cardiac substructure dose and atrial fibrillation risk were included in the structured dosimetric synthesis; because of substantial heterogeneity in cardiac structures, dose metrics, cutoff values, model specifications, and atrial fibrillation definitions, dosimetric effects were not quantitatively pooled.

### 2.6 Quality assessment

The methodological quality of the included studies was assessed using the Newcastle–Ottawa Scale (NOS) (14), which evaluates study selection, comparability between groups, and outcome assessment. Two reviewers independently performed the quality assessment, and discrepancies were resolved through discussion. When necessary, unresolved disagreements were adjudicated by a third reviewer. The quality assessment results were compiled in tabular form and used to inform the interpretation of methodological robustness rather than as the sole criterion for study inclusion or statistical weighting.

### 2.7 Statistical analysis and evidence synthesis

Study characteristics, incidence data, dosimetric effect estimates, and quality assessment results were compiled in tabular form. For studies with extractable atrial fibrillation event counts and analyzable denominators, a logit-transformed random-effects proportion meta-analysis was performed to pool the reported incidence of atrial fibrillation after thoracic radiotherapy. Confidence intervals for individual studies were estimated using the exact binomial method, and forest plots were used to display study-specific and pooled estimates. The primary analysis used the DerSimonian–Laird method as the conventional random-effects model to estimate between-study variance (τ²), from which the pooled incidence and corresponding 95% confidence interval were calculated. The DerSimonian–Laird method was retained as the primary model to maintain consistency with conventional proportion meta-analysis and forest plot presentation; given the small number of included cohorts and substantial heterogeneity, interpretation of the main results also considered prediction intervals and multiple sensitivity analyses. Statistical heterogeneity was assessed using Cochran’s Q test and the I² statistic, and prediction intervals were calculated to reflect the potential range of incidence across different study settings. Additional sensitivity analyses were conducted using restricted maximum likelihood (REML) estimation of τ², Hartung–Knapp confidence interval adjustment for small-sample uncertainty, and a logistic-normal generalized linear mixed model (GLMM) to assess the robustness of the main results to different approaches for estimating between-study variance, handling small-sample uncertainty, and specifying the statistical model. On the basis of clinical and methodological heterogeneity, incidence analyses were stratified into all reported atrial fibrillation cohorts and the main incidence analysis cohorts; cohorts with clear perioperative or surgery-related confounding were interpreted as extended or exploratory evidence.

Structured tables and evidence plots were used to synthesize associations between cardiac and cardiac substructure doses and the risk of atrial fibrillation. Because the dosimetric studies differed substantially in terms of cardiac structures, dose metrics, cutoff values, effect-size measures, model specifications, and atrial fibrillation definitions, dosimetric effect sizes were not quantitatively pooled. Because fewer than 10 cohorts were included in the incidence meta-analysis and substantial clinical and methodological heterogeneity was present across studies, formal statistical tests for small-study effects or publication bias were not performed. All the statistical analyses were performed using R software, version 4.5.1, primarily with the meta package, version 8.5-0, and the metafor package, version 4.8-0.

## 3 Results

### 3.1 Results of the search strategy

**Figure 1:**
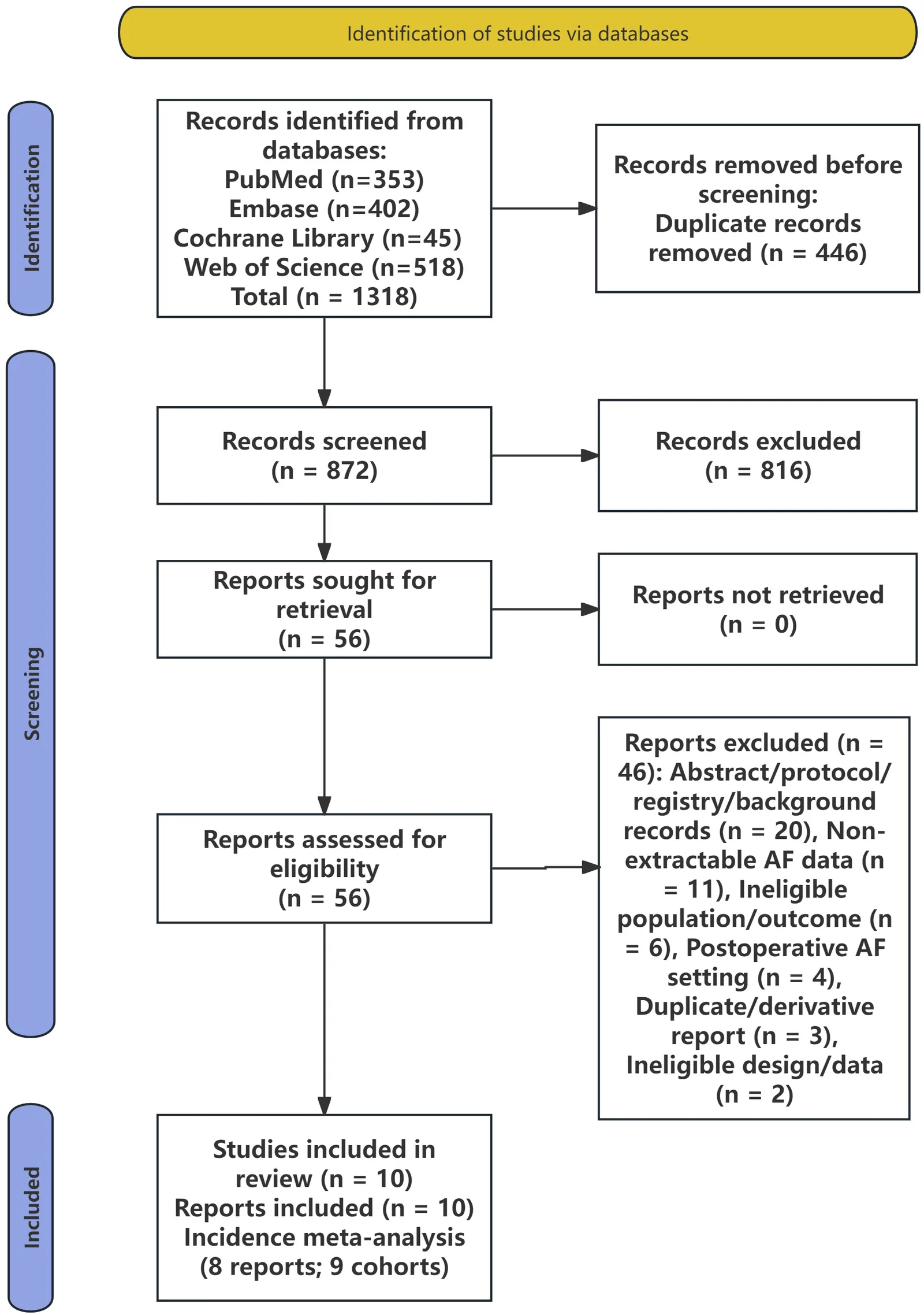
PRISMA flow diagram.

A total of 1,318 records were identified through database searching, including 353 from PubMed, 402 from Embase, 45 from the Cochrane Library, and 518 from Web of Science. After 446 duplicate records were removed, 872 records underwent title and abstract screening, of which 816 were excluded. A total of 56 reports were subsequently assessed for full-text eligibility, of which 46 were excluded for the following reasons: they were conference abstracts, study protocols, registry records, or background-only records (n = 20); atrial fibrillation-specific data could not be extracted (n = 11); the population or outcome did not meet the eligibility criteria (n = 6); they concerned predominantly postoperative atrial fibrillation scenarios not suitable for attribution to thoracic radiotherapy (n = 4); they were duplicate or derivative reports (n = 3); and the study design or data structure did not meet the review requirements (n = 2). Ultimately, 10 studies were included in the systematic review (15–24), of which 8 provided data suitable for single-arm incidence meta-analysis, contributing a total of 9 cohort-level estimates. The study selection process is shown in **Figure 1**.

### 3.2 Description of studies

**Table 1** summarizes the main characteristics of the included studies, including country or region, study period, study design, data source, cancer type and radiotherapy setting, atrial fibrillation outcome definition, handling of preexisting atrial fibrillation, original cohort size, and follow-up duration. Ten studies published between 2022 and 2026 were included (15–24), covering China, Denmark, the Netherlands, the United States, South Korea, and the United Kingdom. Kim 2022 reported two separate cancer cohorts, comprising one for small cell lung cancer and one for non-small cell lung cancer (23); therefore, **Table 1** presents 11 cohort-level rows, while this report was counted as one study in the systematic review. All included studies were observational cohort studies or retrospective analyses based on real-world data. The study populations included non-small cell lung cancer, small cell lung cancer, esophageal squamous cell carcinoma, esophageal cancer, and mixed malignancy cohorts receiving thoracic radiotherapy; the treatment settings included definitive radiotherapy or concurrent chemoradiotherapy, surgery after neoadjuvant chemoradiotherapy, postoperative radiotherapy comparisons, and mixed definitive thoracic radiotherapy cohorts.

**Table 1:** Characteristics of included studies.

Study/cohort	Country/region; study period	Study design/data source	Population and RT setting	AF endpoint/severity	Baseline AF handling/AF risk set	Original cohort n	Reported overall follow-up
Huang 2026 (15) ESCC cohort	China; Apr 2018–Mar 2025	Retrospective single-center cohort; prospectively maintained institutional database	ESCC; standardized nCRT with 41.4 Gy/23 fx; IMRT or VMAT; predominantly followed by esophagectomy	Post-treatment AF; severity not restricted	Prior AF exclusion unclear; strict incident-AF risk set cannot be confirmed	209	28 months (IQR 18–36)
Graven-Nielsen 2026 (16) NSCLC cohort	Denmark; Jan 2010–Dec 2020	Retrospective single-center cohort; electronic health records	NSCLC; curative concurrent chemoradiotherapy with 60–66 Gy/30–33 fx; 3D-CRT or IMRT	*De novo* atrial fibrillation (DNAF); severity not graded	Pre-RT AF excluded; AF risk set n = 247	273	27.4 months (IQR 12.5–57.8)
Qi 2026 (17) ESCC cohort	China; Jan 2019–Dec 2024	Retrospective single-center cohort analysis; prospectively maintained institutional database	ESCC; standardized nCRT with 41.4 Gy/23 fx; IMRT or VMAT; predominantly followed by curative resection	Grade ≥3 AF	Pre-existing arrhythmia not excluded; strict incident-AF risk set cannot be confirmed	358	Not reported
Tohidinezhad 2024 (18) NSCLC cohort	The Netherlands; 2015–2022	Retrospective single-center cohort; institutional clinical and RT dosimetry data	Stage I–oligometastatic stage IV NSCLC; curative-intent conventional photon RT with conventional fractionation or isotoxic dose-escalated schedules	Author-defined new-onset AF; severity not restricted	Baseline AF present in 34/374 (9.1%); recurrent AF after remission included; non-strict *de novo* AF risk set	374	Three-year observation window; median overall follow-up not reported
Butler 2024 (19) Mixed thoracic cohort	USA; 2004–2022	Retrospective single-center cohort; single-institutional database and comprehensive chart review	Primary locoregional NSCLC, breast cancer, Hodgkin lymphoma, or esophageal cancer; definitive thoracic RT	Post-RT clinically significant grade ≥3 AF	Prior grade ≥3 AF before RT in 19/539 (3.5%); excluded from regression analyses; analysis cohort n=520	539	58.8 months (range 0.1–120.0)
Atkins 2024 (20) NSCLC cohort	USA; 1998–2014	Retrospective multicenter cohort; medical record review and dosimetric analysis	Locally advanced NSCLC; 3D-CRT or IMRT	Post-RT grade ≥3 AF	Baseline arrhythmia: 103/748; incident-risk subset n=645	748	5.5 years (Q1–Q3: 3.4–8.1)
Kwon 2024 (21) PORT comparison cohort	South Korea; Jan 2007–Jul 2017	Nationwide population-based retrospective cohort; HIRA claims database	NSCLC after curative surgery and adjuvant chemotherapy; PORT vs non-PORT	New AFib; author-defined clinically significant AFib	Prior AFib excluded; AF risk set n=11,141	11,141	70.6 months (IQR 36.1–101)
Miller 2024 (22) Esophageal cohort	USA; Oct 2007–Dec 2019	Retrospective single-center cohort; chart review and RT dosimetric analysis	Esophageal cancer; curative-intent neoadjuvant, definitive or adjuvant CRT with 41.4–66 Gy in 1.8–2 Gy/fx; 3D-CRT or IMRT; 57.6% underwent surgical resection	Incident grade ≥3 AF after RT initiation	Prior AF in 21/238 (8.8%); excluded from incident-AF analysis; AF risk set n=217	238	22.8 months (IQR 11.0–52.3)
Kim 2022 (23) SCLC cohort	South Korea; Aug 2008–Dec 2019	Retrospective single-center cohort; institutional records and RT dosimetry	Limited-stage SCLC; definitive CRT with etoposide plus platinum; 3D-CRT or IMRT	New-onset AF; severity not graded	Baseline AF present in 3/239 (1.3%); AF-free risk set not separately reported	239	25.7 months (IQR 16.5–47.2)
Kim 2022 (23) NSCLC cohort	South Korea; Aug 2008–Dec 2019	Retrospective single-center cohort; institutional records and RT dosimetry	Locally advanced NSCLC; definitive chemoradiotherapy; predominantly paclitaxel plus carboplatin; 3D-CRT or IMRT	New-onset AF; severity not graded	Baseline AF present in 8/321 (2.5%); AF-free risk set not separately reported	321	36.2 months (IQR 26.9–60.2)
Walls 2024 (24) NSCLC cohort	Northern Ireland, UK; Jan 2015–Dec 2020	Retrospective single-center cohort; oncology, cardiology and death records with RT dosimetric analysis	NSCLC; curative-intent (chemo)RT with 55 Gy/20 fx; 3D-CRT or IMRT, including VMAT	New AF after RT initiation; severe or clinically significant AF; 24 grade 3 and 2 grade 4 events	History of AF excluded; AF risk set n=420	420	Not reported

3D-CRT, three-dimensional conformal radiotherapy; AF/AFib, atrial fibrillation; CRT, chemoradiotherapy; DNAF, *de novo* atrial fibrillation; ESCC, esophageal squamous cell carcinoma; fx, fractions; HIRA, Health Insurance Review and Assessment Service; IMRT, intensity-modulated radiotherapy; IQR, interquartile range; nCRT, neoadjuvant chemoradiotherapy; NSCLC, non-small-cell lung cancer; PORT, postoperative radiotherapy; RT, radiotherapy; SCLC, small-cell lung cancer; VMAT, volumetric modulated arc therapy.

Kim 2022 contributed two cancer-specific cohorts, SCLC and NSCLC, but was counted as one included study in the systematic review. Therefore, **Table 1** presents 11 cohort-level rows derived from 10 included studies. Reported overall follow-up does not necessarily represent the duration or intensity of protocolized AF surveillance; study-specific ascertainment and monitoring details are provided in **Supplementary Table 3**.

There was substantial variation across the included studies in terms of atrial fibrillation outcome definitions and risk-set handling. Some studies reported incident or new-onset atrial fibrillation after excluding preexisting atrial fibrillation; others reported posttreatment atrial fibrillation, author-defined newly diagnosed atrial fibrillation, or clinically significant atrial fibrillation of grade ≥3. Handling of baseline atrial fibrillation was also inconsistent: some cohorts explicitly excluded preexisting atrial fibrillation or prior severe arrhythmia, whereas others incompletely excluded baseline atrial fibrillation, included recurrent atrial fibrillation in patients with prior atrial fibrillation, or did not allow confirmation of a strictly atrial fibrillation-free risk set. **Table 2** summarizes the atrial fibrillation incidence data and analytical classification, including the number of atrial fibrillation events, analyzable denominators, crude incidence, role in evidence synthesis, and major methodological limitations. Adjusted associations between cardiac or cardiac substructure doses and atrial fibrillation risk, together with P values and the main adjustment models, are presented in **Table 3**.

**Table 2:** Incidence data and analytic classification of AF after thoracic radiotherapy.

Study/cohort	AF endpoint/ severity	Baseline AF handling/AF risk set	AF events/risk-set n	Crude incidence	Role in incidence synthesis	Analytic layer/key caveat
Huang 2026 (15) ESCC cohort	Post-treatment AF; severity not restricted	Prior AF exclusion unclear; strict incident-AF risk set cannot be confirmed	25/209	12.0%	Extended/exploratory incidence analysis	Substantial perioperative confounding because most patients underwent esophagectomy
Graven-Nielsen 2026 (16) NSCLC cohort	*De novo* atrial fibrillation (DNAF); severity not graded	Pre-RT AF excluded; AF risk set n = 247	35/247	14.2%	Main incidence analysis	None specific for main analysis
Qi 2026 (17) ESCC cohort	Grade ≥3 AF	Pre-existing arrhythmia not excluded; strict incident-AF risk set cannot be confirmed	Not reported	Not estimable	Narrative dosimetric synthesis only	No extractable event/n for incidence pooling; potential population overlap with Huang 2026
Tohidinezhad 2024 (18) NSCLC cohort	Author-defined new-onset AF; severity not restricted	Baseline AF present in 34/374 (9.1%); recurrent AF after remission included; non-strict *de novo* AF risk set	42/374	11.2%	Main incidence analysis	Risk-set definition was not strictly *de novo*
Butler 2024 (19) Mixed thoracic cohort	Post-RT clinically significant grade ≥3 AF	Prior grade ≥3 AF before RT in 19/539 (3.5%); excluded from regression analyses; analysis cohort n=520	32/520	6.2%	Main incidence analysis	Endpoint restricted to clinically significant grade ≥3 AF
Atkins 2024 (20) NSCLC cohort	Post-RT grade ≥3 AF	Baseline arrhythmia: 103/748; incident-risk subset n=645	56/645 in patients without baseline arrhythmia; original grade ≥3 AF 78/748	8.7%	Main incidence analysis	Endpoint restricted to grade ≥3 AF; incident-risk subset used for pooling
Kwon 2024 (21) PORT comparison cohort	New AFib; author-defined clinically significant AFib	Prior AFib excluded; AF risk set n=11,141	505/11,141	4.5%	Narrative comparative synthesis only	PORT versus non-PORT exposure; not suitable for single-arm incidence pooling
Miller 2024 (22) Esophageal cohort	Incident grade ≥3 AF after RT initiation	Prior AF in 21/238 (8.8%); excluded from incident-AF analysis; AF risk set n=217	51/217	23.5%	Extended/exploratory incidence analysis	Esophageal cancer cohort with substantial surgical/perioperative confounding
Kim 2022 (23) SCLC cohort	New-onset AF; severity not graded	Baseline AF present in 3/239 (1.3%); AF-free risk set not separately reported	9/239	3.8%	Main incidence analysis	AF-free risk set not separately reported
Kim 2022 (23) NSCLC cohort	New-onset AF; severity not graded	Baseline AF present in 8/321 (2.5%); AF-free risk set not separately reported	17/321	5.3%	Main incidence analysis	AF-free risk set not separately reported
Walls 2024 (24) NSCLC cohort	New AF after RT initiation; severe or clinically significant AF; 24 grade 3 and 2 grade 4 events	History of AF excluded; AF risk set n=420	26/420	6.2%	Main incidence analysis	Endpoint reflects severe or clinically significant AF

AF/AFib, atrial fibrillation; DNAF, *de novo* atrial fibrillation; ESCC, esophageal squamous cell carcinoma; PORT, postoperative radiotherapy; RT, radiotherapy; SCLC, small-cell lung cancer; NSCLC, non-small-cell lung cancer.

The incidence meta-analysis included 8 studies contributing 9 cohort-level estimates. Kim 2022 contributed two cancer-specific cohorts, SCLC and NSCLC, but was counted as one included study in the systematic review. The main incidence analysis included 7 cohorts after excluding the two esophageal cancer cohorts with substantial surgical/perioperative confounding. Qi 2026 and Kwon 2024 were retained in the systematic review but were not included in the single-arm incidence meta-analysis.

**Table 3:** Cardiac and cardiac substructure dosimetric associations with AF.

Study/cohort	AF endpoint/severity	Cardiac structure	Dose metric/cutoff	Adjusted estimate	P value	Model/major adjustment
Huang 2026 (15) ESCC cohort	Post-treatment AF; severity not restricted	LIPV	LIPV Dmax >28.87 Gy	adjusted sHR 3.16 (95% CI 1.19–8.42)	P = 0.021	Multivariable competing-risk model; cardiovascular risk factors adjusted; surgical resection treated as a time-dependent covariate
Graven-Nielsen 2026 (16) NSCLC cohort	*De novo* atrial fibrillation (DNAF); severity not graded	SAN	SAN Dmax per Gy	adjusted HR 1.014 (95% CI 1.000–1.029)	P = 0.041	Multivariable Cox regression; adjusted for age, sex, pre-RT arterial hypertension, and pre-RT hemoglobin
		RA	RA Dmax per Gy	adjusted HR 1.017 (95% CI 1.002–1.032)	P = 0.026	
			RA Dmean per Gy	adjusted HR 1.031 (95% CI 1.007–1.056)	P = 0.011	
		Whole heart	MHD per Gy	adjusted HR 1.040 (95% CI 1.006–1.076)	P = 0.021	
			Heart V40Gy per %	adjusted HR 1.024 (95% CI 1.000–1.048)	P = 0.044	
Qi 2026 (17) ESCC cohort	Grade ≥3 AF	Whole heart	Heart Dmax >45.91 Gy	adjusted sHR 3.69 (95% CI 1.59–8.56)	P = 0.0024	Multivariable Fine–Gray competing-risk model; adjusted for age, baseline hypertension, and baseline CHD
Tohidinezhad 2024 (18) NSCLC cohort	Author-defined new-onset AF; severity not restricted	LA	LA Dmax per Gy	adjusted OR 1.022 (95% CI 1.012–1.032)	P < 0.001	Multivariable logistic regression (combined model); age at RT, body mass index, alcohol use, history of cardiac procedures, tumor location, FEV1, creatinine and chemotherapy setting
Butler 2024 (19) Mixed thoracic cohort	Post-RT clinically significant grade ≥3 AF	PV	PV Dmax per 10 Gy	adjusted sHR 1.22 (95% CI 1.14–1.31)	P < 0.001	Fine–Gray model, clustered by cancer type; death as competing risk; adjusted for MAFRS, smoking pack-years, LA volume, LA Dmax and combined PV Dmax
		LA	LA Dmax per Gy	adjusted sHR 0.99 (95% CI 0.99–1.00)	P = 0.21	
Atkins 2024 (20) NSCLC cohort	Post-RT grade ≥3 AF	PV	PV V5 per mL	adjusted sHR 1.04 (95% CI 1.01–1.08)	P = 0.016	Multivariable Fine–Gray competing-risk model; noncardiac death as competing risk; adjusted for age, sex, smoking, alcohol use, baseline arrhythmia/CHD, surgery, chemotherapy type and RT technique
Miller 2024 (22) Esophageal cohort	Incident grade ≥3 AF after RT initiation	LA	LA mean dose per Gy	adjusted HR 1.03 (95% CI 1.01–1.06)	P = 0.022	Multivariable competing-risk regression; death without incident AF as competing risk; adjusted for age and surgical resection
			LA V5 per 5%	adjusted HR 1.16 (95% CI 1.03–1.31)	P = 0.017	
			LA V20 per 5%	adjusted HR 1.10 (95% CI 1.02–1.18)	P = 0.017	
		Whole heart	Whole-heart mean dose per Gy	adjusted HR 1.03 (95% CI 1.00–1.06)	P = 0.094	
Kim 2022 (23) SCLC cohort	New-onset AF; severity not graded	SAN	SAN Dmax ≥53.5 Gy	adjusted HR 14.91 (95% CI 4.00–55.56)	P < 0.001	Multivariable Fine–Gray competing-risk model; the association of SAN Dmax with AF was adjusted for age, alcohol use, number of coronary arteries with calcification, and BMI.
Kim 2022 (23) NSCLC cohort	New-onset AF; severity not graded	SAN	SAN Dmax ≥20.0 Gy	adjusted HR 15.67 (95% CI 2.08–118.20)	P = 0.008	Multivariable Fine–Gray competing-risk model; adjusted for tobacco use, hypertension, cardiovascular disease, and aortic valve calcification
Walls 2024 (24) NSCLC cohort	new AF after RT initiation; severe or clinically significant AF	RPV	RPV V10 per %	adjusted HR 1.01 (95% CI 1.00–1.02)	P = 0.033	Multivariable Fine–Gray competing-risk models; death as competing risk; adjusted for age, sex, heart failure, alcohol consumption, anti-dysrhythmic drug use, chemotherapy, statin therapy, and coronary artery disease
		LPV	LPV V55 per %	adjusted HR 1.02 (95% CI 1.00–1.03)	P = 0.005	

AF, atrial fibrillation; BMI, body mass index; CHD, coronary heart disease; CI, confidence interval; Dmax, maximum dose; Dmean, mean dose; DNAF, *de novo* atrial fibrillation; ESCC, esophageal squamous cell carcinoma; FEV1, forced expiratory volume in 1 second; HR, hazard ratio; LA, left atrium; LIPV, left inferior pulmonary vein; LPV, left pulmonary vein; MAFRS, Mayo AF Risk Score; MHD, mean heart dose; NSCLC, non-small-cell lung cancer; OR, odds ratio; PV, pulmonary vein; RA, right atrium; RPV, right pulmonary vein; RT, radiotherapy; SAN, sinoatrial node; SCLC, small-cell lung cancer; sHR, subdistribution hazard ratio.

Only cardiac or cardiac substructure dosimetric associations were tabulated. Kwon 2024 was not included in this table because it did not report cardiac dosimetry. Butler 2024 also reported left atrial volume as an anatomical covariate, but it is not shown here because it is not a radiation dose metric. Reported P values were extracted when available. Because of heterogeneity in cardiac structures, dose metrics, cutoffs, effect measures, adjustment models, and AF endpoint definitions, these estimates were not pooled quantitatively and were instead summarized as structured dosimetric evidence.

AF ascertainment and surveillance methods also varied substantially across studies (**Supplementary Table 3**). Huang 2026 (15) and Qi 2026 (17) reported scheduled ECG-based surveillance, although Holter monitoring was applied according to study-specific protocols or clinical indications, whereas other studies primarily relied on retrospective medical-record review, standard-of-care ECG or Holter confirmation, or claims-based diagnostic codes. Several studies restricted the endpoint to grade ≥3 or otherwise clinically significant AF. Consequently, the reported overall follow-up duration did not consistently represent a protocolized period of AF monitoring.

### 3.3 Methodological quality assessment

The methodological quality of the included studies was assessed using the Newcastle–Ottawa Scale (NOS), and the results are presented in **Table 4**. NOS scores ranged from 6 to 8 points. As defined by the prespecified NOS threshold, 9 studies were classified as high quality, while 1 was classified as moderate quality. Most studies adequately reported population selection, outcome ascertainment, and adjustment for major confounders; however, some studies had unclear handling of baseline atrial fibrillation, insufficient reporting of follow-up duration, inconsistent definitions of outcome severity, or potential residual confounding. Therefore, the NOS results suggest that the included studies had acceptable methodological quality as observational cohorts, but this should not be interpreted as indicating a high overall certainty of evidence; given that all included studies were observational and showed heterogeneity in atrial fibrillation endpoint definitions, surveillance or ascertainment methods, handling of baseline atrial fibrillation, and dosimetric metrics, the incidence and dosimetric findings should still be interpreted with caution.

**Table 4:** Newcastle–Ottawa Scale quality assessment of included studies.

Study	Selection	Comparability	Outcome	Total NOS score	Overall quality
Huang 2026 (15)	★★★	★★	★★	7	High
Graven-Nielsen 2026 (16)	★★★★	★★	★★	8	High
Qi 2026 (17)	★★★	★★	★	6	Moderate
Tohidinezhad 2024 (18)	★★★	★★	★★	7	High
Butler 2024 (19)	★★★★	★★	★★	8	High
Atkins 2024 (20)	★★★★	★★	★★	8	High
Kwon 2024 (21)	★★★★	★★	★★	8	High
Miller 2024 (22)	★★★★	★	★★	7	High
Kim 2022 (23)	★★★	★★	★★	7	High
Walls 2024 (24)	★★★★	★★	★	7	High

NOS, Newcastle–Ottawa Scale; SCLC, small-cell lung cancer; NSCLC, non-small-cell lung cancer.

The Newcastle–Ottawa Scale evaluates cohort studies across three domains: selection, comparability, and outcome, with a maximum score of 9. Scores of 7–9 were considered high quality, and scores of 5–6 were considered moderate quality. Kim 2022 contributed two cancer-specific cohorts (SCLC and NSCLC), but quality assessment was performed at the study level and therefore counted once.

Each ★ represents one point awarded within the corresponding Newcastle–Ottawa Scale domain. The Selection domain has a maximum of four stars, the Comparability domain a maximum of two stars, and the Outcome domain a maximum of three stars. The total NOS score is the sum of the stars awarded across these three domains, with a maximum score of nine.

### 3.4 Meta-analysis results

#### 3.4.1 Incidence of reported atrial fibrillation after thoracic radiotherapy

**Figure 2:**
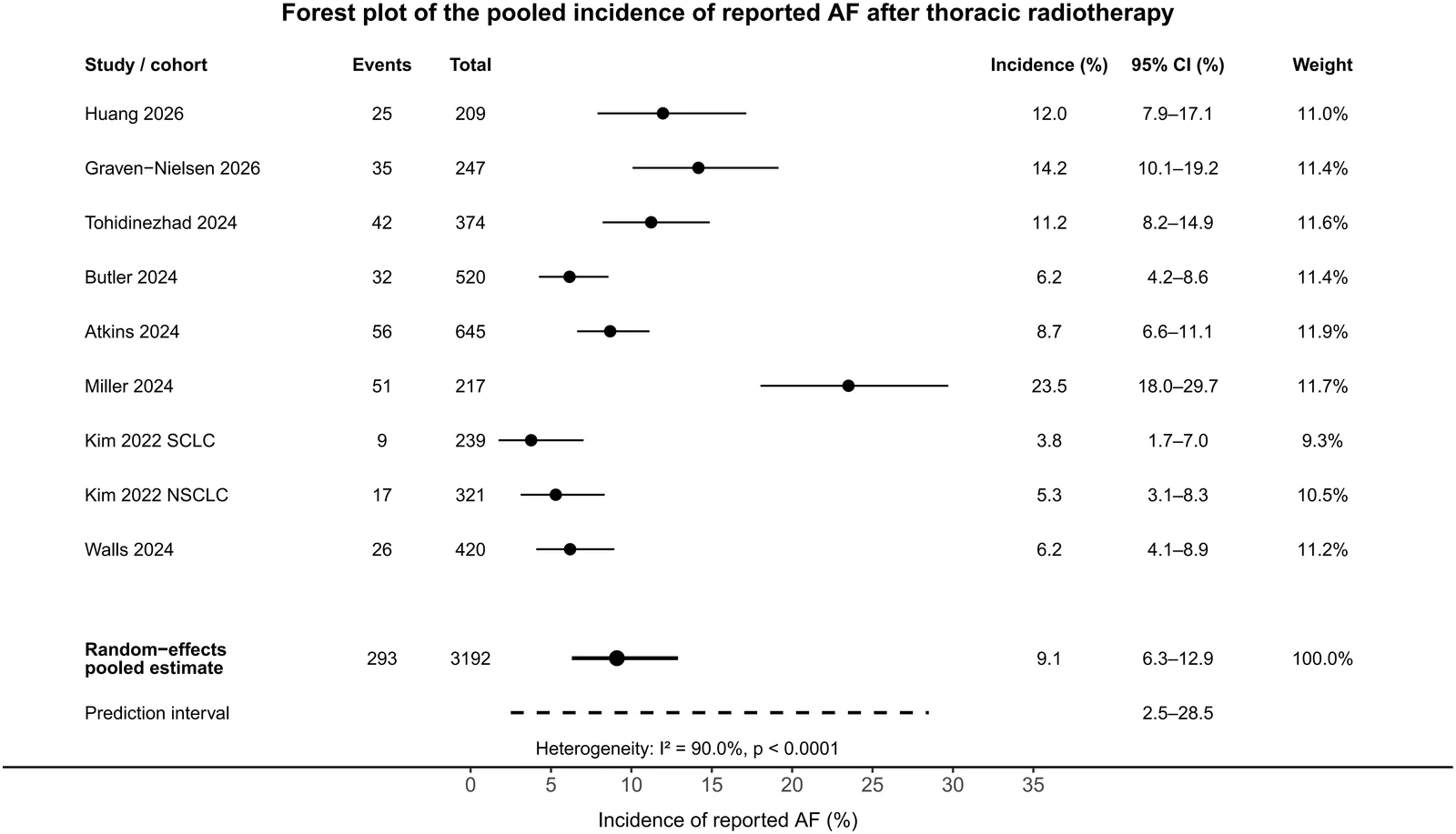
Forest plot of the pooled incidence of reported AF after thoracic radiotherapy. Points indicate cohort-specific or pooled incidence estimates, and horizontal lines indicate 95% confidence intervals. The random-effects pooled estimate was calculated using the conventional DerSimonian-Laird method. The dashed line represents the prediction interval.

Across 9 cohort-level estimates from 8 studies (15, 16, 18–20, 22–24), 3,192 patients receiving thoracic radiotherapy were included, of whom 293 developed study-defined reported atrial fibrillation. The random-effects proportion meta-analysis revealed that the pooled incidence of reported atrial fibrillation after thoracic radiotherapy was 9.1% (95% CI, 6.3%–12.9%; **Figure 2**). This finding suggests that atrial fibrillation is not a rare posttreatment arrhythmic event among patients with malignancies receiving thoracic radiotherapy.

Substantial heterogeneity was observed across studies (I² = 90.0%, τ² = 0.3191; Q = 79.75, df = 8, P < 0.0001), with a wide prediction interval (2.5%–28.5%), suggesting that cancer type, treatment setting, atrial fibrillation definition, event severity, and handling of baseline atrial fibrillation may substantially influence incidence estimates. Therefore, the pooled estimate should be interpreted as the burden of study-defined reported atrial fibrillation across heterogeneous thoracic radiotherapy cohorts rather than as a unified strict incidence estimate of new-onset atrial fibrillation applicable to all patients receiving thoracic radiotherapy.

#### 3.4.2 Main incidence analysis

**Figure 3:**
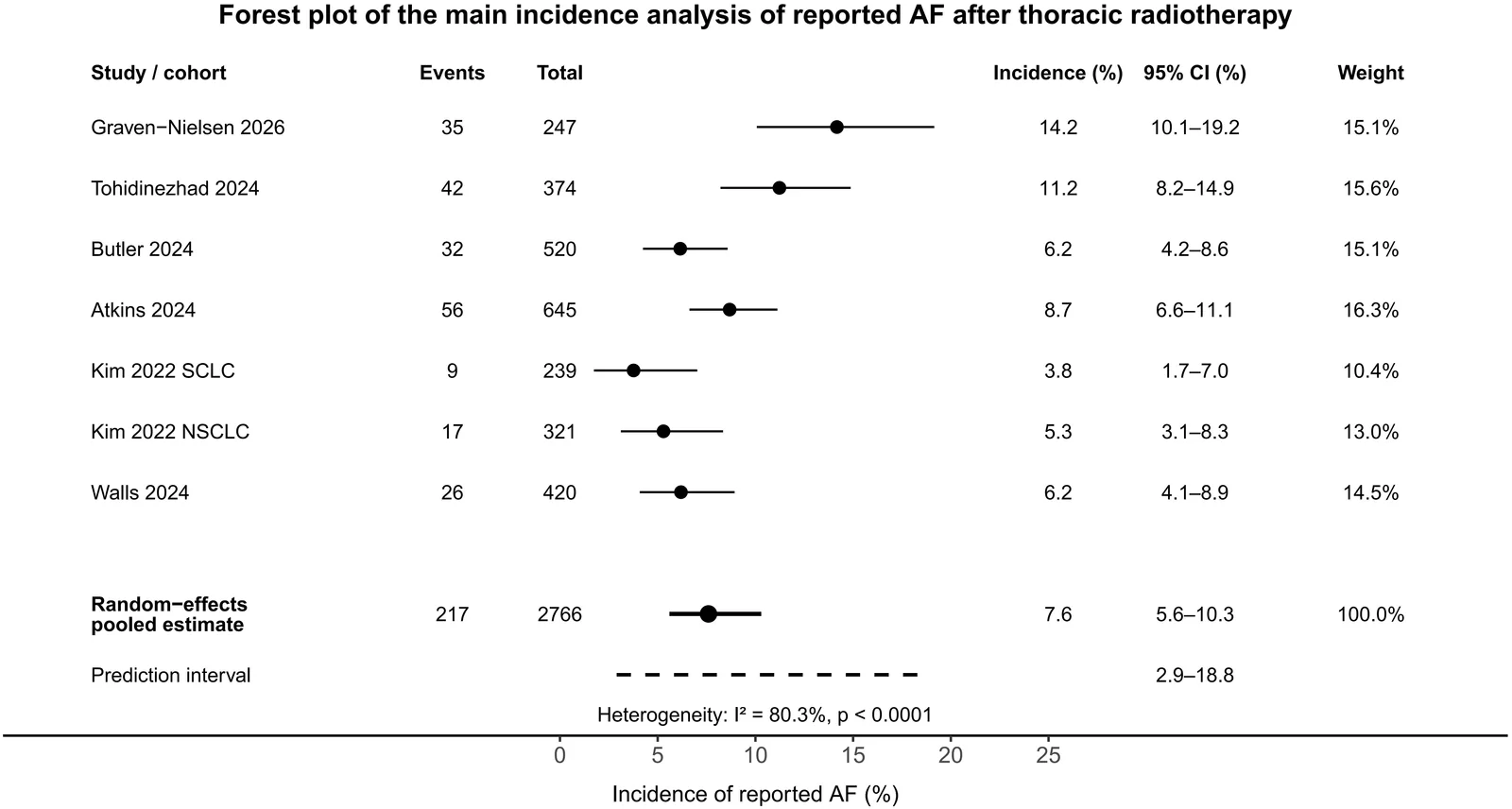
Forest plot of the main incidence analysis of reported AF after thoracic radiotherapy. Points indicate cohort-specific or pooled incidence estimates, and horizontal lines indicate 95% confidence intervals. The random-effects pooled estimate was calculated using the conventional DerSimonian-Laird method. The dashed line represents the prediction interval.

To reduce the influence of perioperative factors related to surgery or trimodality therapy after neoadjuvant chemoradiotherapy for esophageal cancer on atrial fibrillation incidence estimates, the main incidence analysis excluded two esophageal cancer cohorts: Huang 2026 (15) and Miller 2024 (22). This analysis included 7 cohorts from 6 studies (16, 18–20, 23, 24), comprising 2,766 patients, 217 of whom developed study-defined reported atrial fibrillation. The random-effects proportion meta-analysis revealed a 7.6% pooled incidence of reported atrial fibrillation in the main analysis cohorts (95% CI, 5.6%–10.3%; **Figure 3**).

Compared with the overall reported atrial fibrillation analysis, heterogeneity was reduced in the main analysis but remained substantial (I² = 80.3%, τ² = 0.1500; Q = 30.53, df = 6, P < 0.0001), with a prediction interval of 2.9%–18.8%. This finding suggests that cancer type, treatment modality, atrial fibrillation definition, and baseline risk-set handling may still contribute to differences in incidence estimates. Therefore, this estimate is best interpreted as the burden of reported atrial fibrillation in cohorts less affected by perioperative factors or esophageal cancer trimodality therapy, rather than as a uniform incidence estimate that can be directly extrapolated to all patients receiving thoracic radiotherapy.

#### 3.4.3 Sensitivity analyses

Across sensitivity analyses using alternative random-effects approaches, the pooled incidence point estimates remained generally stable. In the overall reported atrial fibrillation analysis, the pooled incidence estimates from the DerSimonian–Laird primary model, restricted maximum likelihood model, Hartung–Knapp-adjusted model, and logistic-normal generalized linear mixed model were 9.10%, 9.08%, 9.08%, and 8.95%, respectively. In the main incidence analysis cohorts, the corresponding pooled incidence estimates were 7.60%, 7.58%, 7.58%, and 7.48%, respectively. The 95% confidence intervals overlapped across models, indicating that the main findings were robust to alternative methods for estimating between-study variance, confidence interval adjustment for small-sample uncertainty, and statistical model specifications (**Supplementary Figure 1**).

#### 3.4.4 Narrative evidence from the PORT comparison cohort

Kwon 2024 (21) was a cohort study comparing postoperative radiotherapy (PORT) with non-PORT and was therefore not included in the single-arm incidence meta-analysis. This study revealed that PORT was not significantly associated with an increased risk of new-onset atrial fibrillation in patients with non-small cell lung cancer who underwent curative surgery and adjuvant chemotherapy (adjusted odds ratio (OR), 0.96; 95% confidence interval (CI), 0.66–1.37; P = 0.806). Because this study did not report heart or cardiac substructure dosimetric metrics and differed in design from the single-arm thoracic radiotherapy incidence cohorts, it was synthesized as comparative narrative evidence.

### 3.5 Dosimetric associations involving the heart and cardiac substructures

**Figure 4:**
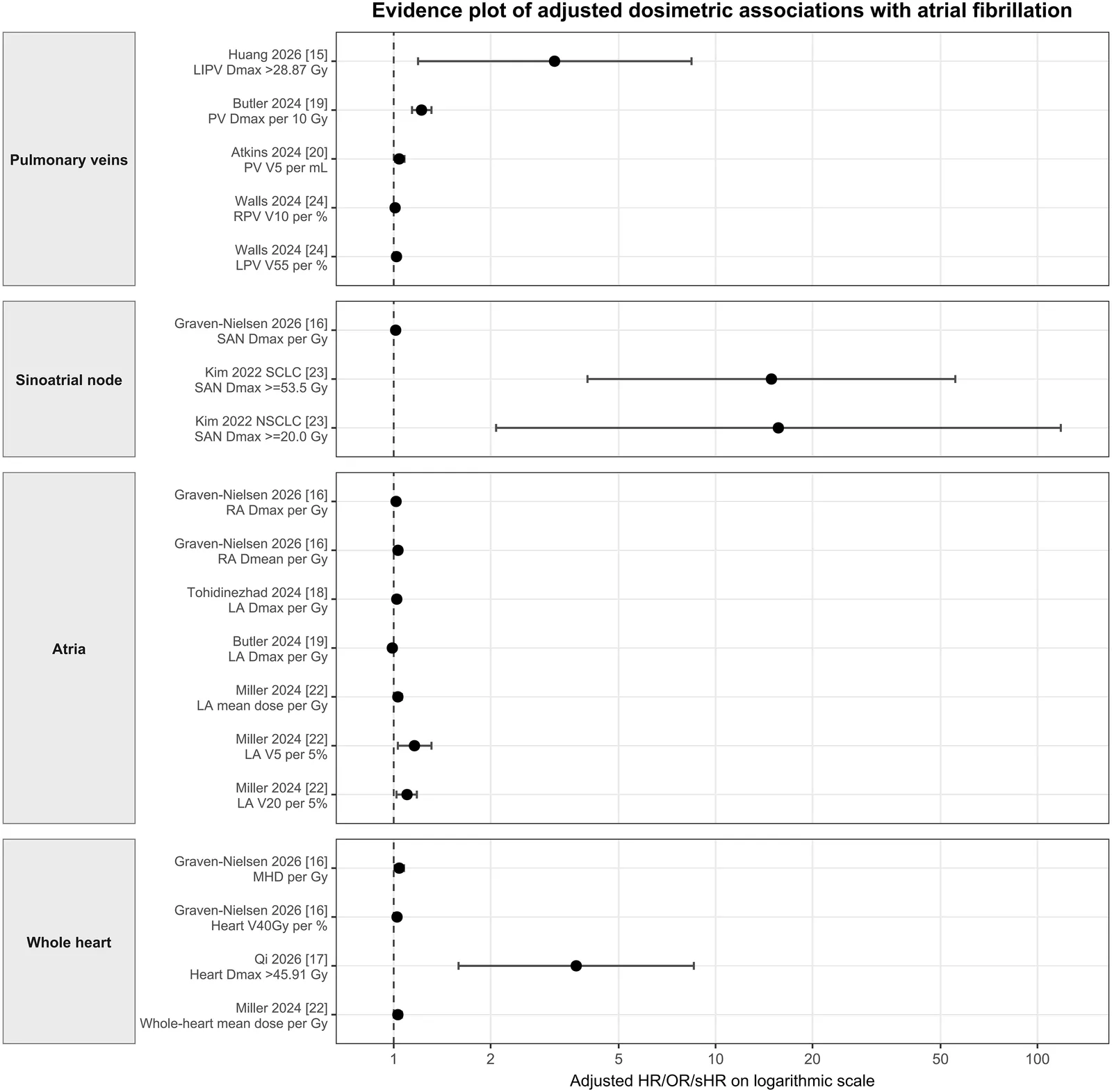
Evidence plot of adjusted dosimetric associations with atrial fibrillation. Points indicate adjusted effect estimates, and horizontal lines indicate 95% confidence intervals. Effect measures include hazard ratios, odds ratios, and subdistribution hazard ratios, as reported in the original studies. Estimates are displayed on a logarithmic scale and grouped by cardiac structure. Exact estimates, P values, and adjustment models are provided in **Table 3**. Because effect measures, dose units, and cutoffs differ across studies, the horizontal positions should be interpreted as the direction and precision of each reported association, not as directly comparable effect magnitudes across rows. Because of heterogeneity in cardiac structures, dose metrics, cutoffs, effect measures, adjustment models, and AF endpoint definitions, these estimates were not pooled quantitatively.

Nine reports (15–20, 22–24) provided information on the associations between cardiac or cardiac substructure doses and the risk of atrial fibrillation, involving structures such as the pulmonary veins, sinoatrial node, left atrium, right atrium (RA), and whole heart. Among these reports, Huang 2026 and Qi 2026 both involved esophageal squamous cell carcinoma cohorts treated with neoadjuvant chemoradiotherapy, with potential overlap in source populations (15, 17). Therefore, the dosimetric evidence was synthesized at the report-signal level and was not interpreted as independent replication across 9 completely separate study populations. Because studies differed substantially in terms of atrial fibrillation outcome definitions, dose–volume metrics, cutoff values, effect-size measures, statistical models, and adjusted covariates, dosimetric effect sizes were not quantitatively pooled but were summarized using structured tables and evidence plots (**Table 3**; **Figure 4**).

Cardiac substructure delineation and dosimetric extraction methods were also heterogeneous across the studies (**Supplementary Table 4**). The workflows included expert manual contouring, automated or deep-learning segmentation with manual verification, and mixed approaches. In several studies, the sinoatrial node (SAN) or atrioventricular node (AVN) was represented by an atlas-based or spherical surrogate rather than a directly visualized anatomical structure. Quality-assurance procedures ranged from formal inter- and intraobserver reproducibility assessments to review of a small random sample. Dmax was not operationalized uniformly: Graven-Nielsen 2026 (16) used D0.1cc for the SAN and D1cc for the RA; Kim 2022 (23) used D0.035cc, whereas several other studies did not specify a near-maximum volume definition.

Overall, candidate dosimetric signals related to pulmonary vein dose were repeatedly observed across multiple reports, although pulmonary vein segmentation, dose metrics, event severity, and statistical models were inconsistent. Huang 2026 (15) reported that a left inferior pulmonary vein Dmax >28.87 Gy was associated with an increased risk of posttreatment atrial fibrillation; Butler 2024 (19) reported that each 10-Gy increase in pulmonary vein Dmax was associated with a higher risk of clinically significant atrial fibrillation of grade ≥3; Atkins 2024 (20) and Walls 2024 (24) also suggested that low- or high-dose pulmonary vein volume metrics were associated with grade ≥3 or new-onset atrial fibrillation. Dose metrics for the sinoatrial node also merit attention, although the associated evidence remains uncertain. Kim 2022 (23) reported that a higher sinoatrial node Dmax was associated with new-onset atrial fibrillation risk in both small cell lung cancer and non-small cell lung cancer cohorts, although the confidence intervals around the effect estimates were wide; Graven-Nielsen 2026 (16) suggested that each 1-Gy increase in sinoatrial node Dmax may be associated with *de novo* atrial fibrillation risk, but the lower bound of the confidence interval was close to 1.00.

Selected atrial dose metrics showed candidate dosimetric signals; in contrast, evidence regarding the whole-heart dose was weaker and less consistent. Graven-Nielsen 2026 (16) reported that the right atrial Dmax and right atrial Dmean were associated with the risk of *de novo* atrial fibrillation and suggested that the mean heart dose and heart V40 Gy may also be related to this risk. Tohidinezhad 2024 (18) suggested that the left atrial Dmax was associated with the risk of author-defined new-onset atrial fibrillation, whereas Miller 2024 (22) suggested that the left atrial mean dose and V5/V20 were associated with the risk of incident grade ≥3 atrial fibrillation. Regarding whole-heart dose metrics, Qi 2026 (17) reported that a whole-heart Dmax >45.91 Gy was associated with an increased risk of grade ≥3 atrial fibrillation, and Graven-Nielsen 2026 (16) suggested that selected whole-heart dose metrics may be related to the risk of atrial fibrillation; however, Miller 2024 (22) did not find a statistically significant association between the whole-heart mean dose and AF risk (P = 0.094). Overall, the available evidence suggests that atrial fibrillation after thoracic radiotherapy may be associated with dose exposure to the pulmonary veins, sinoatrial node, atria, and selected whole-heart parameters. However, because of substantial between-study heterogeneity and inconsistent findings for the whole-heart dose, these findings should be regarded as structured dosimetric signals rather than uniform dose–response effects suitable for direct quantitative pooling.

## 4 Discussion

This systematic review and meta-analysis suggests that study-defined reported atrial fibrillation is a clinically relevant event among patients with malignancies receiving thoracic radiotherapy. In the main incidence analysis, which excluded cohorts substantially affected by perioperative factors or esophageal cancer trimodality therapy, 217 cases of reported atrial fibrillation occurred among 2,766 patients across 7 cohorts, yielding a pooled incidence of 7.6%. When all 9 cohorts eligible for single-arm incidence synthesis were included, 293 of 3,192 patients developed reported atrial fibrillation, corresponding to a pooled incidence of 9.1%. Sensitivity analyses using alternative random-effects methods yielded similar point estimates, supporting the robustness of the main incidence estimates across different statistical approaches. Nevertheless, the included studies differed substantially in terms of atrial fibrillation definitions, handling of baseline atrial fibrillation, event severity, treatment pathways, ascertainment methods, and follow-up duration. Importantly, overall clinical follow-up was not equivalent to continuous or protocolized rhythm surveillance. Scheduled ECG and Holter monitoring could identify asymptomatic or paroxysmal AF that might be missed by routine records, symptom-triggered testing, or administrative coding, whereas grade ≥3 endpoints preferentially captured clinically significant events. Differences in surveillance intensity and endpoint severity may have contributed to the observed heterogeneity. Therefore, these estimates should be interpreted as the study-defined burden of reported atrial fibrillation across heterogeneous detection pathways rather than as a uniform estimate of strict *de novo* atrial fibrillation incidence under standardized monitoring.

The distinction between the main and extended analyses has important methodological and clinical implications. The crude incidence of reported atrial fibrillation was relatively high in the two esophageal cancer cohorts, but both were substantially influenced by factors related to surgery or trimodality therapy (15, 22). In Huang 2026, most patients underwent esophagectomy after neoadjuvant chemoradiotherapy, and a substantial proportion of atrial fibrillation events occurred postoperatively (15). Miller 2024 also reported incident atrial fibrillation events during the early postoperative period after esophagectomy (22). These events may reflect the combined influence of radiation-related cardiac or cardiac substructure dose exposure, oncological treatment context, and perioperative stress. Postoperative atrial fibrillation may arise from inflammatory responses, autonomic disturbances, changes in volume status, infection, anemia, and surgical stress, thereby limiting the ability to attribute AF to radiation-related cardiac dose exposure alone. Accordingly, the main incidence analysis provides a more conservative estimate in cohorts less affected by perioperative pathways, whereas the extended analysis reflects the broader real-world reported burden. Consistent with this cautious interpretation, the nationwide PORT cohort study by Kwon et al. revealed no significant association between postoperative radiotherapy and newly diagnosed atrial fibrillation after adjustment among patients with non-small cell lung cancer who underwent curative surgery and adjuvant chemotherapy (adjusted OR, 0.96; 95% CI, 0.66–1.37; P = 0.806) (21).

Residual confounding by disease severity and treatment geometry also remains important. Larger, more central, or anatomically advanced tumors may increase cardiac exposure while also identifying patients with greater systemic illness, pulmonary compromise, treatment intensity, hospitalization, or perioperative stress. Target volume, mediastinal proximity, surgery, infection, hypoxia, anemia, and systemic therapy may therefore influence both cardiac dose and the risk of AF. Adjustment strategies were inconsistent across studies and did not uniformly account for these factors. Accordingly, adjusted dose-AF associations should be interpreted as hypothesis-generating rather than as evidence of a direct causal effect of radiation.

The dosimetric synthesis provides a hypothesis-generating evidence map rather than a basis for clinical dose constraints. Existing adjusted models suggest that radiation dose to the pulmonary veins, atria, and sinoatrial node as well as selected whole-heart metrics may be associated with the risk of atrial fibrillation after thoracic radiotherapy (15–20, 22–24). Because potential source-population overlap may exist among some esophageal squamous cell carcinoma reports (15, 17), these findings should not be interpreted as independent validation across completely separate populations. Relevant signals included pulmonary vein maximum-dose and volume-based metrics, such as PV Dmax, PV V5, RPV V10, LPV V55, and LIPV Dmax; atrial dose metrics, such as LA Dmax, LA mean dose, LA V5/V20, and RA Dmax/Dmean; conduction system–related metrics, such as SAN Dmax; and whole-heart dose metrics, such as whole-heart Dmax, mean heart dose, heart Dmax, and heart V40 Gy. These associations are biologically plausible because pulmonary vein myocardial sleeves are important triggers of atrial fibrillation, whereas atrial electrical remodeling, structural remodeling, inflammation, and fibrosis contribute to the formation and maintenance of the atrial fibrillation substrate (25–27). Radiation may contribute to these processes through oxidative stress, microvascular injury, inflammation, fibrosis, autonomic nervous system alterations, and conduction tissue injury. Moreover, contemporary research on radiation-associated cardiac injury has increasingly shifted from whole-heart dose assessment toward modeling associations between cardiac substructure doses and specific cardiac endpoints (28–30).

Interpretation of individual cardiac substructure dose metrics is further limited by delineation uncertainty and nonuniform dose definitions. Small or anatomically indistinct structures such as the SAN and pulmonary vein regions were represented using manual atlas-based contours, automated contours, or geometric surrogates across studies. Dmax was also operationalized differently, including point or near-maximum definitions, and may be particularly sensitive to contour shifts, dose-grid resolution, and steep local dose gradients. In contrast, mean whole-heart dose is generally less sensitive to small contour variations but provides less anatomical specificity. Accordingly, reported substructure-specific dose metrics and thresholds should be interpreted as candidate dosimetric signals rather than directly transferable planning constraints.

These findings suggest that atrial fibrillation after thoracic radiotherapy should not be interpreted solely through conventional whole-heart dose metrics such as the mean heart dose or whole-heart Vx. Because atrial fibrillation differs from ischemic events, pericarditis, and heart failure in terms of both mechanism and clinical phenotype, future studies should consider the atria, pulmonary veins, and conduction system–related structures as candidate dosimetric regions. Such studies should evaluate whether these structures provide independent or incremental predictive information after adjustment for traditional atrial fibrillation risk factors, baseline cardiovascular disease, systemic therapy exposure, surgical or perioperative factors, and competing mortality. At present, these structures are better suited for standardized delineation and reporting in prospective trials, registry studies, and real-world cohorts than for immediate translation into mandatory radiotherapy planning constraints (31–33).

In clinical practice, the present findings are most relevant to risk-adapted monitoring and collaborative cardio-oncology management. Patients receiving thoracic radiotherapy often have established risk factors for atrial fibrillation, including advanced age, hypertension, coronary artery disease, smoking exposure, chronic lung disease, systemic therapy exposure, and preexisting cardiovascular disease. Recent cardio-oncology consensus statements and prospective studies have increasingly emphasized individualized cardiovascular risk assessment, optimization of modifiable risk factors, and stratified follow-up before, during, and after cancer treatment; early cardio-oncology interventions and post-radiotherapy atrial fibrillation screening strategies are also being evaluated in prospective research settings (34–39). For patients expected to receive higher doses to the atria, pulmonary veins, or sinoatrial node and who also have a high clinical risk of atrial fibrillation, baseline electrocardiographic assessment, optimization of modifiable risk factors, education regarding atrial fibrillation–related symptoms, and symptom-triggered or risk-adapted rhythm monitoring may be reasonable. These approaches should be prioritized for evaluation within research, registry, or risk-stratified follow-up frameworks, particularly when atrial fibrillation may influence anticoagulation decisions, systemic therapy continuity, surgical timing, hospitalization risk, or the overall treatment pathway.

This study has several limitations. First, this review was not prospectively registered in a public database; therefore, the stratified, sensitivity, and exploratory analyses should be interpreted with caution. Second, all included studies were observational, and most used retrospective designs; residual confounding, selection bias, and outcome surveillance bias cannot be fully excluded. In particular, confounding by disease severity, treatment geometry, systemic therapy, surgery, or acute illness could not be eliminated. Third, the studies differed substantially in terms of atrial fibrillation definitions, severity grading, ascertainment methods, surveillance intensity, and handling of baseline atrial fibrillation; in some cohorts, it could not be confirmed that the risk set consisted strictly of patients without prior atrial fibrillation. Overall follow-up could not be assumed to represent continuous AF surveillance, and asymptomatic or paroxysmal AF may have been differentially detected across studies. Fourth, follow-up duration, handling of death as a competing risk, systemic therapy exposure, and surgical treatment pathways varied across studies, particularly in esophageal cancer cohorts undergoing surgery after neoadjuvant chemoradiotherapy (15, 22). Fifth, dosimetric studies were heterogeneous in structural definitions, delineation and verification methods, dose metrics, cutoffs, effect-size measures, and covariate models, precluding quantitative pooling of dose–response effects. Maximum-dose definitions were not uniform across studies, and several small cardiac substructures were represented by atlas-based, automated, or spherical surrogate approaches, introducing additional measurement uncertainty. Finally, the number of included studies was limited, restricting the assessment of publication bias, and potential source-population overlap may exist among some esophageal squamous cell carcinoma reports (15, 17). For these reasons, we adopted a conservative analytic classification and avoided repeated inclusion of potentially overlapping data within the same pooled analysis.

## 5 Conclusion

This systematic review and meta-analysis shows that study-defined reported atrial fibrillation after thoracic radiotherapy represents a non-negligible clinical burden, with pooled incidences of 7.6% in the main incidence analysis and 9.1% across all cohorts eligible for single-arm incidence synthesis. Existing adjusted dosimetric evidence suggests that the pulmonary veins, atria, sinoatrial node, and selected whole-heart dose metrics may represent candidate dosimetric regions associated with atrial fibrillation after thoracic radiotherapy. However, because the current evidence is derived mainly from heterogeneous observational studies, these findings should be interpreted as hypothesis-generating rather than as definitive causal evidence or clinical dose constraints. Future research should validate these dosimetric signals through standardized atrial fibrillation definitions, rhythm-monitoring protocols, reproducible cardiac substructure delineation, prospective multicenter studies, and prespecified adjustment for clinical and treatment-related confounders, and evaluate their value for cardioprotective radiotherapy planning and risk-adapted monitoring.

## Data Availability

The original contributions presented in the study are included in the article/**Supplementary Material**. Further inquiries can be directed to the corresponding authors.
