# Supplementary material for: Associations between reported atrial fibrillation burden and cardiac substructure dosimetric variables in patients receiving thoracic radiotherapy for malignancies involving the thorax: a systematic review and meta-analysis
Source: Front Oncol. 2026 Sep 10;16:1910673. doi: 10.3389/fonc.2026.1910673 (PMC13600814; doi:10.3389/fonc.2026.1910673)
Supplement: Supplementary file 1 [file DataSheet1.docx]

**Figure S1.** Sensitivity analyses of pooled reported AF incidence across random-effects methods.

Points indicate pooled incidence estimates, and horizontal lines indicate 95% confidence intervals. The DerSimonian-Laird method was used as the conventional primary random-effects model. REML, Hartung–Knapp correction, and logistic-normal GLMM were used as sensitivity analyses to evaluate the robustness of the primary incidence estimates.
