# Supplementary material for: Associations between reported atrial fibrillation burden and cardiac substructure dosimetric variables in patients receiving thoracic radiotherapy for malignancies involving the thorax: a systematic review and meta-analysis
Source: Front Oncol. 2026 Sep 10;16:1910673. doi: 10.3389/fonc.2026.1910673 (PMC13600814; doi:10.3389/fonc.2026.1910673)
Supplement: Supplementary file 2 [file Table1.docx]

**Supplementary Table 1.** Representative PubMed search strategy

The literature search was conducted in PubMed, Embase, the Cochrane Library, and Web of Science. The final search was performed on May 17, 2026. No language, publication-date, or article-type restrictions were applied during the database searches. Search strategies were adapted according to the syntax requirements of each database. The PubMed search strategy is shown as a representative example.

| Database | Search query |
| --- | --- |
| PubMed | (("Atrial Fibrillation"[Mesh] OR "atrial fibrillation"[Title/Abstract] OR AF[Title/Abstract] OR "atrial arrhythmia"[Title/Abstract] OR "atrial arrhythmias"[Title/Abstract] OR "supraventricular arrhythmia"[Title/Abstract] OR "supraventricular arrhythmias"[Title/Abstract] OR "cardiac arrhythmia"[Title/Abstract] OR "cardiac arrhythmias"[Title/Abstract]) AND ("Radiotherapy"[Mesh] OR radiotherapy[Title/Abstract] OR "radiation therapy"[Title/Abstract] OR irradiation[Title/Abstract] OR "thoracic radiotherapy"[Title/Abstract] OR "thoracic radiation"[Title/Abstract] OR "chest radiotherapy"[Title/Abstract] OR "chest radiation"[Title/Abstract]) AND (thoracic[Title/Abstract] OR lung[Title/Abstract] OR pulmonary[Title/Abstract] OR mediastinal[Title/Abstract] OR breast[Title/Abstract] OR esophageal[Title/Abstract] OR oesophageal[Title/Abstract] OR lymphoma[Title/Abstract] OR "non-small cell lung cancer"[Title/Abstract] OR NSCLC[Title/Abstract] OR "small cell lung cancer"[Title/Abstract] OR SCLC[Title/Abstract])) NOT (animals[Mesh] NOT humans[Mesh]) |
| PubMed supplementary dosimetric search | (("atrial fibrillation"[Title/Abstract] OR "atrial arrhythmia"[Title/Abstract] OR "supraventricular arrhythmia"[Title/Abstract] OR arrhythmia[Title/Abstract] OR arrhythmias[Title/Abstract]) AND (radiotherapy[Title/Abstract] OR "radiation therapy"[Title/Abstract] OR irradiation[Title/Abstract]) AND ("cardiac substructure"[Title/Abstract] OR "cardiac substructures"[Title/Abstract] OR "heart dose"[Title/Abstract] OR "mean heart dose"[Title/Abstract] OR "cardiac dose"[Title/Abstract] OR "left atrium"[Title/Abstract] OR "right atrium"[Title/Abstract] OR "left atrial dose"[Title/Abstract] OR "right atrial dose"[Title/Abstract] OR "sinoatrial node"[Title/Abstract] OR "sinus node"[Title/Abstract] OR "pulmonary vein"[Title/Abstract] OR "pulmonary veins"[Title/Abstract] OR dosimetric[Title/Abstract] OR dosimetry[Title/Abstract] OR V5[Title/Abstract] OR V10[Title/Abstract] OR V20[Title/Abstract] OR V30[Title/Abstract])) |
