# Supplementary material for: Associations between reported atrial fibrillation burden and cardiac substructure dosimetric variables in patients receiving thoracic radiotherapy for malignancies involving the thorax: a systematic review and meta-analysis
Source: Front Oncol. 2026 Sep 10;16:1910673. doi: 10.3389/fonc.2026.1910673 (PMC13600814; doi:10.3389/fonc.2026.1910673)
Supplement: Supplementary file 3 [file Table2.docx]

| **Section and Topic** | **Item #** | **Checklist item** | **Location where item is reported** |
| --- | --- | --- | --- |
| **TITLE** | | |  |
| Title | 1 | Identify the report as a systematic review. | Page 1, Title, lines 1–3 |
| **ABSTRACT** | | |  |
| Abstract | 2 | See the PRISMA 2020 for Abstracts checklist. | Page 1–2, Abstract, lines 24–50 |
| **INTRODUCTION** | | |  |
| Rationale | 3 | Describe the rationale for the review in the context of existing knowledge. | Page 2–3, Introduction, lines 56–88 |
| Objectives | 4 | Provide an explicit statement of the objective(s) or question(s) the review addresses. | Page 3, Introduction, lines 89–98 |
| **METHODS** | | |  |
| Eligibility criteria | 5 | Specify the inclusion and exclusion criteria for the review and how studies were grouped for the syntheses. | Page 4–5, Sections 2.3 and 2.5, lines 131–147 and 168–185 |
| Information sources | 6 | Specify all databases, registers, websites, organisations, reference lists and other sources searched or consulted to identify studies. Specify the date when each source was last searched or consulted. | Page 4, Section 2.2, lines 114–127 |
| Search strategy | 7 | Present the full search strategies for all databases, registers and websites, including any filters and limits used. | Page 4, Section 2.2, lines 114–127; Supplementary Table 1 provides the representative PubMed search strategy. |
| Selection process | 8 | Specify the methods used to decide whether a study met the inclusion criteria of the review, including how many reviewers screened each record and each report retrieved, whether they worked independently, and if applicable, details of automation tools used in the process. | Page 4, Section 2.3, lines 131–147; Page 7, Section 3.1, lines 233–245 |
| Data collection process | 9 | Specify the methods used to collect data from reports, including how many reviewers collected data from each report, whether they worked independently, any processes for obtaining or confirming data from study investigators, and if applicable, details of automation tools used in the process. | Page 5, Section 2.4, lines 151–164 |
| Data items | 10a | List and define all outcomes for which data were sought. Specify whether all results that were compatible with each outcome domain in each study were sought (e.g. for all measures, time points, analyses), and if not, the methods used to decide which results to collect. | Page 4, Section 2.3, lines 134–138; Page 5, Section 2.4, lines 153–164 |
|  | 10b | List and define all other variables for which data were sought (e.g. participant and intervention characteristics, funding sources). Describe any assumptions made about any missing or unclear information. | Page 5, Section 2.4, lines 153–164; Tables 1–3 |
| Study risk of bias assessment | 11 | Specify the methods used to assess risk of bias in the included studies, including details of the tool(s) used, how many reviewers assessed each study and whether they worked independently, and if applicable, details of automation tools used in the process. | Page 6, Section 2.6, lines 189–195; Table 4 |
| Effect measures | 12 | Specify for each outcome the effect measure(s) (e.g. risk ratio, mean difference) used in the synthesis or presentation of results. | Page 5, Section 2.4, lines 158–164; Page 6–7, Section 2.7, lines 199–224 |
| Synthesis methods | 13a | Describe the processes used to decide which studies were eligible for each synthesis (e.g. tabulating the study intervention characteristics and comparing against the planned groups for each synthesis (item #5)). | Page 5–6, Section 2.5, lines 168–185 |
|  | 13b | Describe any methods required to prepare the data for presentation or synthesis, such as handling of missing summary statistics, or data conversions. | Page 5–7, Sections 2.5 and 2.7, lines 168–185 and 199–224 |
|  | 13c | Describe any methods used to tabulate or visually display results of individual studies and syntheses. | Page 6–7, Section 2.7, lines 199–224; Figures 2–4; Tables 1–4 |
|  | 13d | Describe any methods used to synthesize results and provide a rationale for the choice(s). If meta-analysis was performed, describe the model(s), method(s) to identify the presence and extent of statistical heterogeneity, and software package(s) used. | Page 6–7, Section 2.7, lines 199–229 |
|  | 13e | Describe any methods used to explore possible causes of heterogeneity among study results (e.g. subgroup analysis, meta-regression). | Page 5–7, Sections 2.5 and 2.7, lines 168–185 and 217–220; Page 9, lines 303–309; Page 10, lines 320–327 |
|  | 13f | Describe any sensitivity analyses conducted to assess robustness of the synthesized results. | Page 6, Section 2.7, lines 213–217; Page 10, Section 3.4.3, lines 331–340; Figure S1 |
| Reporting bias assessment | 14 | Describe any methods used to assess risk of bias due to missing results in a synthesis (arising from reporting biases). | Page 7, Section 2.7, lines 225–229; formal statistical tests for small-study effects/publication bias were not performed |
| Certainty assessment | 15 | Describe any methods used to assess certainty (or confidence) in the body of evidence for an outcome. | Page 6, Section 2.6, lines 189–195. Methodological quality was assessed using the Newcastle–Ottawa Scale; no formal GRADE assessment was performed. |
| **RESULTS** | | |  |
| Study selection | 16a | Describe the results of the search and selection process, from the number of records identified in the search to the number of studies included in the review, ideally using a flow diagram. | Page 7, Section 3.1, lines 233–245; Figure 1 |
|  | 16b | Cite studies that might appear to meet the inclusion criteria, but which were excluded, and explain why they were excluded. | Page 7, Section 3.1, lines 236–242; Figure 1 |
| Study characteristics | 17 | Cite each included study and present its characteristics. | Page 7–8, Section 3.2, lines 249–275; Table 1 |
| Risk of bias in studies | 18 | Present assessments of risk of bias for each included study. | Page 8, Section 3.3, lines 279–291; Table 4 |
| Results of individual studies | 19 | For all outcomes, present, for each study: (a) summary statistics for each group (where appropriate) and (b) an effect estimate and its precision (e.g. confidence/credible interval), ideally using structured tables or plots. | Page 9–11, Sections 3.4–3.5, lines 297–396; Tables 2–3; Figures 2–4 |
| Results of syntheses | 20a | For each synthesis, briefly summarise the characteristics and risk of bias among contributing studies. | Page 8–10, Sections 3.3–3.4, lines 279–340; Tables 1–2 and 4 |
|  | 20b | Present results of all statistical syntheses conducted. If meta-analysis was done, present for each the summary estimate and its precision (e.g. confidence/credible interval) and measures of statistical heterogeneity. If comparing groups, describe the direction of the effect. | Page 9–10, Sections 3.4.1–3.4.3, lines 297–340; Figures 2–3; Figure S1 |
|  | 20c | Present results of all investigations of possible causes of heterogeneity among study results. | Page 9–10, Sections 3.4.1–3.4.2, lines 303–327; Page 12, Discussion, lines 414–431 |
|  | 20d | Present results of all sensitivity analyses conducted to assess the robustness of the synthesized results. | Page 10, Section 3.4.3, lines 331–340; Figure S1 |
| Reporting biases | 21 | Present assessments of risk of bias due to missing results (arising from reporting biases) for each synthesis assessed. | Page 7, Section 2.7, lines 225–229; Page 13, Discussion, lines 490–491 |
| Certainty of evidence | 22 | Present assessments of certainty (or confidence) in the body of evidence for each outcome assessed. | Page 8, Section 3.3, lines 279–291; Page 13, Discussion, lines 478–494. Certainty of evidence was discussed narratively; no formal GRADE assessment was performed. |
| **DISCUSSION** | | |  |
| Discussion | 23a | Provide a general interpretation of the results in the context of other evidence. | Page 11–13, Discussion, lines 400–477 |
|  | 23b | Discuss any limitations of the evidence included in the review. | Page 13, Discussion, lines 478–494 |
|  | 23c | Discuss any limitations of the review processes used. | Page 13, Discussion, lines 478–494 |
|  | 23d | Discuss implications of the results for practice, policy, and future research. | Page 13, Discussion, lines 451–477; Page 14, Conclusion, lines 498–509 |
| **OTHER INFORMATION** | | |  |
| Registration and protocol | 24a | Provide registration information for the review, including register name and registration number, or state that the review was not registered. | Page 3, Section 2.1, lines 103–108; the review was not prospectively registered |
|  | 24b | Indicate where the review protocol can be accessed, or state that a protocol was not prepared. | Page 3, Section 2.1, lines 105–108; an internal protocol was developed but was not publicly available |
|  | 24c | Describe and explain any amendments to information provided at registration or in the protocol. | Not applicable; the review was not prospectively registered and no public protocol amendments were made |
| Support | 25 | Describe sources of financial or non-financial support for the review, and the role of the funders or sponsors in the review. | Page 14, Funding, lines 528–531 |
| Competing interests | 26 | Declare any competing interests of review authors. | Page 15, Conflict of interest, lines 533–536 |
| Availability of data, code and other materials | 27 | Report which of the following are publicly available and where they can be found: template data collection forms; data extracted from included studies; data used for all analyses; analytic code; any other materials used in the review. | Page 14, Data availability statement, lines 511–516; Page 19, Supplementary Material, lines 736–739 |

*From:*  Page MJ, McKenzie JE, Bossuyt PM, Boutron I, Hoffmann TC, Mulrow CD, et al. The PRISMA 2020 statement: an updated guideline for reporting systematic reviews. BMJ 2021;372:n71. doi: 10.1136/bmj.n71. This work is licensed under CC BY 4.0. To view a copy of this license, visit <https://creativecommons.org/licenses/by/4.0/>
