# Supplementary material for: Associations between reported atrial fibrillation burden and cardiac substructure dosimetric variables in patients receiving thoracic radiotherapy for malignancies involving the thorax: a systematic review and meta-analysis
Source: Front Oncol. 2026 Sep 10;16:1910673. doi: 10.3389/fonc.2026.1910673 (PMC13600814; doi:10.3389/fonc.2026.1910673)
Supplement: Supplementary file 4 [file Table3.docx]

| **Study / cohort** | **AF endpoint and baseline AF handling** | **Ascertainment source / diagnostic criteria** | **Surveillance and adjudication** | **Observation period / overall follow-up** | **Key ascertainment considerations** |
| --- | --- | --- | --- | --- | --- |
| Huang 2026(15)  ESCC cohort | Post-treatment AF; no severity restriction was specified. Baseline ECG was performed, but explicit exclusion of pre-existing AF was not reported | 12-lead ECG and Holter monitoring; AF required absolutely irregular RR intervals, absent discernible P waves, and duration ≥30 s according to the 2016 ESC criteria | Routine ECG every 3 months; immediate 24-h Holter for symptoms; 48-h Holter every 6 months in asymptomatic patients. Two blinded cardiologists interpreted ECGs, with discrepancies resolved by a third senior electrophysiologist | Median follow-up 28 months (IQR 18–36); median time to AF 13 months; 2-year cumulative incidence 8.89% | Active surveillance detected 7 asymptomatic events among 25 AF events; prior-AF exclusion was unclear, and 15/25 events occurred after esophagectomy |
| Graven-Nielsen 2026(16) NSCLC cohort | De novo AF after RT; severity not graded. Pre-RT AF was excluded; AF risk set n=247 | Electronic health-record review before and after RT; specific ECG/Holter criteria were not reported | Cardiac outcomes were identified from medical records. No protocolized rhythm-monitoring schedule or independent event-adjudication process was reported | Median observation 27.4 months (IQR 12.5–57.8); median time to DNAF 16 months (IQR 6.4–39.4); DNAF probability 9.3% at 1 year and 12.2% at 2 years | Retrospective record-based ascertainment may have missed asymptomatic or paroxysmal AF; surveillance practices may also have varied over the 10-year study period |
| Qi 2026(17) ESCC cohort | Grade ≥3 AF after nCRT, classified according to CTCAE v4.0. Pre-existing arrhythmia was present in 76/358 patients and was not excluded; prior-AF status and a strict incident-AF risk set were not reported | Structured EMR abstraction integrating ECG reports, cardiology consultation notes, and arrhythmia-specific medication records; no AF-specific ECG duration or rhythm criterion was reported | Routine 12-lead ECG at baseline, during nCRT, preoperatively, and at each follow-up visit; 24-h Holter for symptoms or abnormal ECG findings. Follow-up visits occurred every 3 months for the first 2 years; adjudicator identity, blinding, and monitoring beyond 2 years were not reported | Follow-up was measured from nCRT initiation; median overall follow-up was not reported in the main text. Median time to first AF was 12 months (95% CI 11–14), and the 2-year cumulative incidence was 8.89% | Scheduled ECG surveillance was used, but strict incident AF status cannot be confirmed. Most patients underwent esophagectomy, and surgery was modeled as a binary rather than time-dependent covariate, leaving possible residual postoperative confounding |
| Tohidinezhad 2024(18) NSCLC cohort | New-onset AF was defined as the first documented AF episode in patients without prior AF; recurrent AF after remission was also included. Baseline AF was present in 34/374 (9.1%), resulting in a non-strict de novo AF risk set | Baseline and follow-up ECGs; only diagnoses confirmed by treating cardiologists based on ECG or Holter monitoring were counted. No minimum episode duration or specific rhythm criteria were reported | Follow-up ECGs were reviewed, but their timing and frequency and any symptom-triggered monitoring protocol were not reported. Independent adjudication and blinding were not reported | AF was assessed during up to 3 years of follow-up; 42/374 patients developed AF. Median overall follow-up and AF-specific monitoring duration were not reported | Cardiologist-confirmed ECG/Holter ascertainment strengthened diagnostic specificity, but the absence of a protocolized monitoring schedule may have missed asymptomatic or paroxysmal AF. Inclusion of recurrent AF after remission broadened the endpoint beyond strictly incident AF |
| Butler 2024(19) Mixed thoracic cohort | Grade ≥3 AF after RT according to CTCAE v5.0; new-onset AF alone was considered insufficient unless accompanied by clinically significant features. Prior AF was present in 19/539 patients (3.5%); these patients were excluded from regression, spline, and ROC analyses, leaving 520 patients for incident-AF analyses | Comprehensive chart review of physician notes, diagnostic testing, imaging reports, and shared external electronic medical records. AF events were identified by two investigators under the supervision of a fellowship-trained cardiologist. No AF-specific ECG/Holter duration or rhythm criteria were reported | No protocolized rhythm-monitoring schedule was reported, and continuous monitoring for asymptomatic AF was explicitly not feasible. Formal independent adjudication and blinding of outcome reviewers to dosimetry were not reported | Median follow-up 58.8 months (range 0.1–120.0); median time to AF 22.9 months (range 0.3–120). The 5-year cumulative AF incidence was 5.2% overall. Time-to-AF follow-up began at RT completion, with censoring at the last follow-up or at the start of reirradiation | The grade ≥3 endpoint favored reliable retrospective identification of clinically significant AF but excluded asymptomatic and grade 1–2 events. Of 35 post-RT events, 32 occurred in patients without prior AF; descriptive counts included three prior-AF patients, whereas incident-risk analyses excluded patients with prior AF |
| Atkins 2024(20) NSCLC cohort | Grade ≥3 AF according to CTCAE v4.0. Pre-existing arrhythmia was present in 103/748 patients (13.8%) and was not excluded; prior AF was not reported separately, so a strict incident-AF risk set could not be confirmed | Detailed manual electronic medical record review incorporating standard-of-care ECG reports and cardiology notes. No AF-specific rhythm or episode-duration criteria were reported | No protocolized ECG or ambulatory rhythm-monitoring schedule was reported. Reviewer identity, independent adjudication, disagreement resolution, and blinding to dosimetry were not reported | Follow-up was calculated from RT initiation. Median follow-up was 5.5 years (Q1–Q3, 3.4–8.1); 78 AF events occurred, with median time to AF of 2.0 years (Q1–Q3, 0.9–4.2) and 2-year cumulative incidence of 8.0% | The grade ≥3 endpoint focused on clinically significant AF but may have undercaptured asymptomatic or milder events. Because patients with baseline arrhythmia were retained and accounted for 22 of 78 AF events, the outcome did not represent a strictly defined incident-AF population |
| Kwon 2024(21) PORT comparison cohort | New AF was defined by ICD-10 code I48 recorded during hospitalization or on at least two outpatient visits. Patients with AF before the index date were excluded. A separate grade ≥2 AF endpoint required AF-related medication for ≥90 days or an AF-related procedure or operation | Nationwide HIRA claims data incorporating diagnosis, prescription, procedure, treatment, and hospitalization codes. ECG/Holter confirmation, rhythm criteria, episode duration, and physician adjudication were not available | No protocolized rhythm surveillance was performed or reported. AF ascertainment depended on health care encounters and qualifying claims; no independent clinical adjudication was reported | Follow-up began at the lung-cancer diagnosis date. Median follow-up was 70.6 months (IQR 36.1–101), with administrative censoring on July 31, 2022. The 5-year cumulative incidence of new AF was 4.1%, and the mean interval to AF diagnosis was 24 months | Prior AF was excluded, supporting a claims-defined incident-AF cohort. However, AF ascertainment depended on coded clinical encounters and may have missed undiagnosed or minimally managed episodes. Because follow-up began at diagnosis rather than PORT initiation, early postoperative or chemotherapy-associated AF could contribute to the outcome |
| Miller 2024(22) Esophageal cohort | Incident grade ≥3 AF after RT initiation in patients without prior AF. Prior AF was present in 21/238 patients (8.8%) and excluded from the incident-AF risk set; 7 of these patients subsequently developed grade ≥3 symptomatic AF | Manual review of oncology, cardiology, emergency-department, and hospital records, including ECG, telemetry, ambulatory rhythm monitoring, and cardiac imaging. Events were graded using CTCAE v5.0. No AF-specific episode-duration or rhythm criterion was reported | All cardiac events were adjudicated independently by two cardiologists blinded to heart and cardiac-substructure RT dose. No protocolized ECG or ambulatory-monitoring schedule was reported, and rigorous arrhythmia assessment was not routinely performed at all follow-up visits. | Follow-up began at RT initiation. Median follow-up was 22.8 months (IQR 11.0–52.3); median time to incident AF was 4.1 months (IQR 2.9–7.3), and 1-year cumulative incidence was 19.5% | Independent blinded cardiologist adjudication strengthened event classification, but asymptomatic AF may have been missed. Of 51 incident-AF events, 25 occurred within 30 days of surgery; surgery therefore remained an important potential confounder, although analyses excluding early postoperative AF were performed |
| Kim 2022(23) SCLC and  NSCLC cohorts | New-onset AF after RT initiation was determined by comparing baseline and follow-up ECGs. Baseline AF was present in 3/239 SCLC and 8/321 NSCLC patients; the article did not explicitly state whether these patients were excluded from all time-to-event risk-set denominators | In-depth medical-record review by two independent cardiologists, with comparison of baseline and follow-up ECGs. No AF-specific rhythm criteria, minimum episode duration, or ambulatory-monitoring requirement were reported | Patients underwent clinical follow-up every 2–4 months for the first 3 years and every 6 months for the next 2 years. Cardiac events were reviewed by two independent cardiologists blinded to dosimetric data. Patients were not routinely screened for arrhythmias | Time to cardiac events was measured from RT initiation. Overall median follow-up was 32.7 months (IQR 22.1–56.6); cohort-specific follow-up was 25.7 months (IQR 16.5–47.2) for SCLC and 36.2 months (IQR 26.9–60.2) for NSCLC. Nine and 17 new-onset AF events occurred, respectively | Blinded review by two cardiologists and baseline-to-follow-up ECG comparison strengthened event classification, but the absence of routine arrhythmia screening may have missed asymptomatic or subclinical AF |
| Walls 2024(24)  NSCLC cohort | New AF after RT initiation, graded using CTCAE v5.0. Patients with a history of AF were excluded. All 26 AF events were grade ≥3, including 24 grade 3 and 2 grade 4 events | Oncology records were reviewed for baseline and subsequent cardiovascular outcomes, and AF events were verified by an electrophysiology subspecialist cardiologist. AF-specific ECG criteria, minimum episode duration, and ambulatory-monitoring requirements were not reported | No protocolized ECG or ambulatory rhythm-monitoring schedule was reported. Events were verified by one electrophysiology cardiologist; independent adjudication by multiple reviewers and blinding to dosimetric data were not reported | Time to AF was measured from RT initiation until death or last follow-up. Median overall follow-up was not reported. Median OS was 21.8 months (IQR 10.8–35.1), and median time to AF was 13.3 months (IQR 8.4–13.3) | Exclusion of prior AF supported an incident-AF cohort, but the grade ≥3 endpoint primarily captured clinically significant events and may have missed asymptomatic or less severe AF. Overall follow-up duration and AF-specific monitoring intensity were not reported separately |
