# Supplementary material for: Associations between reported atrial fibrillation burden and cardiac substructure dosimetric variables in patients receiving thoracic radiotherapy for malignancies involving the thorax: a systematic review and meta-analysis
Source: Front Oncol. 2026 Sep 10;16:1910673. doi: 10.3389/fonc.2026.1910673 (PMC13600814; doi:10.3389/fonc.2026.1910673)
Supplement: Supplementary file 5 [file Table4.docx]

| **Study / cohort** | **Structures and imaging / planning dataset** | **Delineation method** | **Operators, blinding, and quality assurance** | **Dose-metric operational definition** | **Key methodological considerations** |
| --- | --- | --- | --- | --- | --- |
| Huang 2026(15) ESCC cohort | Four individual PVs on contrast-enhanced planning CT with 3-mm slices | Manual contouring from the PV–LA junction to the first branch bifurcation using a published PV approach | Experienced radiation oncologist; outcome blinding not reported. Dosimetric data were independently verified by two individuals | Dmax, Dmean, and V5–V40 were extracted in Eclipse or Pinnacle; Dmax was described as the local maximum, without a specified near-maximum volume | Formal contour reproducibility was not reported, and respiratory- and cardiac-motion-related dose variation was not fully corrected; Dmax-based cutoffs may therefore be contour-sensitive |
| Graven-Nielsen 2026(16) NSCLC cohort | Heart, RA, and SAN on planning CT with 2.0- or 2.5-mm slices using ARIA v18.0 | Manual SAN delineation using an RT-planning atlas; manual RA delineation using Danish national cardiac-contouring guidelines; whole-heart contours derived from clinical RT planning | Two independent investigators; inter- and intra-observer reproducibility was assessed in 10 randomly selected cases using repeat delineation after 1 week and ICCs of structure-specific dose metrics. SAN and RA contours were validated by a cardiac-CT specialist cardiologist and a thoracic oncologist; blinding was not reported | MHD and heart V25Gy/V40Gy; RA Dmean and Dmax (defined as D1cc); SAN Dmean and Dmax (defined as D0.1cc) | Formal reproducibility testing supported dose-metric reliability; however, the SAN was anatomically localized rather than directly visualized, and its small size may introduce interobserver contouring and interfraction positional uncertainty |
| Qi 2026(17) ESCC cohort | SAN, AVN, and whole heart on radiotherapy planning CT; slice thickness, contrast use, and treatment-planning system were not reported | Manual atlas-based delineation was used for the SAN and AVN. The SAN was modeled as a 2-cm-diameter sphere along the right atrial crista terminalis between the venae cavae, tangent to the right atrial epicardium and centered at the level of the ascending-aorta origin. The AVN was modeled as a 2-cm-diameter sphere within Koch’s triangle at the atrioventricular septal junction, 1 cm above the most cranial CT slice showing the left atrium. The whole-heart delineation method was not reported | SAN and AVN were manually contoured by X.Y. and H.Z.; operator training, allocation or consensus procedures, outcome blinding, expert verification, and formal inter-/intra-observer reproducibility testing were not reported | Dmax, Dmean, and V5–V40 were calculated for the SAN, AVN, and whole heart; no near-maximum volume definition was provided for Dmax | Fixed 2-cm spherical surrogates may not fully reflect patient-specific nodal anatomy. Formal contour reproducibility testing and the near-maximum volume underlying Dmax were not reported, limiting assessment of contour and dose-metric robustness |
| Tohidinezhad 2024(18) NSCLC cohort | Whole heart, four chambers, SAN, and AVN were derived from 4D planning CT. Intravenous contrast was used at the radiation oncologist’s discretion, and FDG-PET/CT was additionally incorporated for patients with nodal involvement. Treatment planning used Eclipse v11 | The four cardiac chambers were automatically segmented using an open-source deep-learning model. The SAN and AVN were geometrically defined as 10-mm-radius spheres positioned using other cardiac substructure contours. The whole heart was manually contoured by treating radiation oncologists | Automatic contours were reviewed to identify misleading segmentations that could affect dose-volume extraction; the authors reported that the contours appeared satisfactory on visual inspection | Whole heart and chambers: volume, Dmin, Dmean, Dmax, and V5–V60 in 5-Gy increments; SAN and AVN: Dmin, Dmean, and Dmax. No near-maximum volume definition was reported for Dmax | The authors acknowledged that auto-contouring inaccuracies could introduce dosimetric uncertainty. Visual review was performed, but quantitative contour validation and reproducibility testing were not reported; geometric nodal surrogates may not fully reflect patient-specific anatomy |
| Butler 2024(19) Mixed thoracic cohort | Four individual PVs, with an additional supernumerary right middle-lobe PV when present, as well as the LA, SAN, combined left coronary arteries, and whole-heart dose metrics were evaluated on RT planning CT. Dosimetry was assessed in MIM v7.3.3; 72/539 patients lacked DICOM-format RT dose data | Cardiac-substructure contours were manually created in MIM. Individual PVs were delineated using a published PV atlas and combined for the primary analysis; SAN and combined left coronary arteries were contoured according to published guidelines. Detailed LA and whole-heart contouring protocols were not reported | Two physicians, blinded to clinical outcomes, created the contours. A random 5% sample was audited by a third physician, followed by research-group consensus. Formal quantitative inter-/intra-observer reproducibility metrics were not reported | Doses were converted to EQD2 using α/β = 3. Mean dose and Dmax were calculated for PVs, LA, and SAN; PV and LA analyses emphasized Dmax, described as a single-point maximum. Combined left coronary artery V15 was the absolute volume receiving ≥15 Gy. No near-maximum volume definition was reported for Dmax | Blinded contouring and audit of a random 5% sample provided basic quality control, but quantitative reproducibility was not reported. Because PV contours are surrogates for CT-invisible myocardial sleeves and vary with branching anatomy and the cardiac cycle, single-point Dmax may be contour-sensitive. Missing DICOM dose data in 72 patients reduced the dosimetric cohort |
| Atkins 2024(20) NSCLC cohort | Cardiac chambers, coronary arteries, PVs, left-ventricular myocardium, SAN, and AVN were evaluated using RT planning CT and dose-plan data. Dose-plan information was available for 701/748 patients; CT slice thickness and contrast use were not reported | Chambers and coronary arteries were manually segmented. PVs and left-ventricular myocardium were segmented using TotalSegmentator. SAN and AVN were generated using separate nnU-Net models trained on atlas-guided manual contours from an independent 46-patient NSCLC dataset; outputs were converted to 1-cm-radius spheres | Manual and AI-generated contours underwent manual verification by listed investigators. SAN/AVN model development used training, validation, and test sets of 36, 5, and 5 patients. Outcome blinding, quantitative segmentation performance, and formal inter-/intra-observer reproducibility were not reported | For all structures, Dmean, Dmax, and V5–V60 in 5-Gy increments were calculated, with Vx expressed as both percentage and milliliters. No near-maximum volume definition was reported for Dmax | Manual verification provided quality control, but quantitative contour accuracy and reproducibility were not reported. The SAN/AVN models were trained and tested on a small independent dataset, and fixed 1-cm-radius spherical outputs may not fully represent patient-specific nodal anatomy. Missing dose-plan data reduced the dosimetric cohort to 701 patients |
| Miller 2024(22) Esophageal cohort | Whole heart and four chambers—LA, RA, LV, and RV—on radiotherapy planning CT. Free-breathing CT was used for treatment planning, with 4D CT generally used for motion assessment in lower-esophageal/GEJ tumors. Contouring and DVH analysis were performed in Eclipse. CT slice thickness and contrast use were not reported | The whole heart and four chambers were manually contoured on planning CT by one investigator and independently reviewed and edited by a second investigator using the Feng cardiac atlas | Initial manual contouring by one investigator, followed by independent review and editing by a second. Contourer blinding and formal reproducibility testing were not reported; cardiac-event adjudicators were blinded to cardiac dose | Whole-heart and chamber Dmean, Dmax, and V5–V50 were calculated in Eclipse. No near-maximum volume definition was reported for Dmax | Atlas-guided delineation with independent secondary review provided contour quality control, but quantitative reproducibility was not reported. Free-breathing planning CT with 4D motion assessment used only in selected patients may leave residual motion-related uncertainty. The absence of a near-maximum volume definition also limits assessment of Dmax robustness |
| Kim 2022(23) SCLC and  NSCLC cohorts | Patient-specific restorable RT plans were analyzed. Structures included the RA, RV, LA, LV, LAD, RCA, LCx, SAN, and AVN. The main article did not report CT acquisition parameters, contrast use, treatment-planning system, or dose-calculation algorithm | RA, RV, LA, LV, LAD, RCA, and LCx were delineated according to a cardiac atlas using an in-house deep-learning autosegmentation tool. SAN and AVN were manually delineated according to a cardiac conduction-node atlas | The auto-segmented contours were reviewed by three radiation oncologists blinded to clinical factors. The operators and review process for manual SAN/AVN delineation were not reported. Quantitative segmentation performance and formal inter-/intra-observer reproducibility were also not reported in the main article | Dmax was defined as the minimum dose to the hottest 0.035 cc. Dmean and V5–V60 in 5-Gy increments were also calculated, with Vx expressed as the percentage of the structure receiving at least the indicated dose | Three-radiation-oncologist review provided quality control for the auto-segmented chamber and coronary contours. The explicit 0.035-cc near-maximum definition improved interpretability of Dmax. However, quantitative contour accuracy and reproducibility were not reported, and the operator and verification procedures for the manually delineated SAN and AVN remained unclear |
| Walls 2024(24)  NSCLC cohort | Contrast-enhanced 4D planning CT was used, with diagnostic PET fused for target and organ-at-risk delineation. PVs, SAN, and LA were assessed in Eclipse plans generated using 3DCRT, IMRT, or VMAT. Superior and inferior PVs were combined by side as RPV and LPV structures. CT slice thickness was not reported | PVs and SAN were manually segmented by a clinical oncologist using published contouring references. The LA was auto-contoured using a validated open-source deep-learning algorithm | A clinical oncologist performed the manual PV and SAN segmentation. A random 5% sample of cases underwent verification by a radiation oncologist. Blinding, quantitative contour accuracy, and formal inter-/intra-observer reproducibility were not reported | DVHs were calculated using the AAA v16.1.0 algorithm. Preselected PV metrics were V10, Dmean, and V55; SAN Dmax and LA V20 were also assessed. No near-maximum volume definition was reported for SAN Dmax | Random verification of 5% of cases provided limited contour quality control, but comprehensive independent review and quantitative reproducibility were not reported. Combining superior and inferior PVs by side streamlined analysis but reduced individual-vein resolution |
